# Subclinical *in utero* Zika virus infection is associated with interferon alpha sequelae and sex-specific molecular brain pathology in asymptomatic porcine offspring

**DOI:** 10.1371/journal.ppat.1008038

**Published:** 2019-11-14

**Authors:** Ivan Trus, Daniel Udenze, Brian Cox, Nathalie Berube, Rebecca E. Nordquist, Franz Josef van der Staay, Yanyun Huang, Gary Kobinger, David Safronetz, Volker Gerdts, Uladzimir Karniychuk

**Affiliations:** 1 Vaccine and Infectious Disease Organization-International Vaccine Centre (VIDO-InterVac), University of Saskatchewan, Saskatoon, Canada; 2 School of Public Health, University of Saskatchewan, Saskatoon, Canada; 3 Department of Physiology, Department of Obstetrics and Gynaecology, University of Toronto, Toronto, ON, Canada; 4 Behavior and Welfare Group, Department of Farm Animal Health, Faculty of Veterinary Medicine, Utrecht University, Utrecht, CL, Netherlands; 5 Brain Center Rudolf Magnus, Utrecht University, Utrecht, Netherlands; 6 Prairie Diagnostic Services, Saskatoon, Canada; 7 CHUL and Laval University, Québec City, QC, Canada; 8 Canada National Microbiology Laboratory, Public Health Agency of Canada, Winnipeg, MB, Canada; 9 Department of Veterinary Microbiology, Western College of Veterinary Medicine, University of Saskatchewan, Saskatoon, Canada; NIH, UNITED STATES

## Abstract

Zika virus (ZIKV) infection during human pregnancy may lead to severe fetal pathology and debilitating impairments in offspring. However, the majority of infections are subclinical and not associated with evident birth defects. Potentially detrimental life-long health outcomes in asymptomatic offspring evoke high concerns. Thus, animal models addressing sequelae in offspring may provide valuable information. To induce subclinical infection, we inoculated selected porcine fetuses at the mid-stage of development. Inoculation resulted in trans-fetal virus spread and persistent infection in the placenta and fetal membranes for two months. Offspring did not show congenital Zika syndrome (e.g., microcephaly, brain calcifications, congenital clubfoot, arthrogryposis, seizures) or other visible birth defects. However, a month after birth, a portion of offspring exhibited excessive interferon alpha (IFN-α) levels in blood plasma in a regular environment. Most affected offspring also showed dramatic IFN-α shutdown during social stress providing the first evidence for the cumulative impact of prenatal ZIKV exposure and postnatal environmental insult. Other eleven cytokines tested before and after stress were not altered suggesting the specific IFN-α pathology. While brains from offspring did not have histopathology, lesions, and ZIKV, the whole genome expression analysis of the prefrontal cortex revealed profound sex-specific transcriptional changes that most probably was the result of subclinical *in utero* infection. RNA-seq analysis in the placenta persistently infected with ZIKV provided independent support for the sex-specific pattern of *in utero*-acquired transcriptional responses. Collectively, our results provide strong evidence that two hallmarks of fetal ZIKV infection, altered type I IFN response and molecular brain pathology can persist after birth in offspring in the absence of congenital Zika syndrome.

## Author summary

A number of studies showed that Zika virus (ZIKV) can cause severe abnormalities in fetuses, e.g., brain lesions, and subsequently life-long developmental and cognitive impairment in children. However, the majority of infections in pregnant women are subclinical and are not associated with developmental abnormalities in fetuses and newborns. It is known that disruptions to the *in utero* environment during fetal development can program increased risks for disease in adulthood. For this reason, children affected *in utero* even by mild ZIKV infection can appear deceptively healthy at birth but develop immune dysfunction and brain abnormalities during postnatal development. Here, we used the porcine model of subclinical fetal ZIKV infection to determine health sequelae in offspring which did not show apparent signs of the disease. We demonstrated that subclinical fetal infection was associated with abnormal immunological responses in apparently healthy offspring under normal environmental conditions and during social stress. We also showed silent sex-specific brain pathology as represented by altered gene expression. Our study provides new insights into potential outcomes of subclinical *in utero* ZIKV infection. It also emphasizes that further attempts to better understand silent pathology and develop alleviative interventions in ZIKV-affected offspring should take into account interactions of host factors, like sex, and environmental insults, like social stress.

## Introduction

Zika virus (ZIKV) infection during human pregnancy may lead to fetal death, brain lesions, *in utero* growth restriction, and microcephaly in newborns resulting in severe life-long impairments [1–4]. Critically, the majority of congenital infections in humans is subclinical [2,5] and is not associated with easily identifiable brain lesions or birth defects. Deleterious and less severe delayed neurodevelopmental, motor, and neurosensory abnormalities in apparently normal at birth human offspring have been described later within one-two years of life [6,7]. Potentially detrimental life-long health outcomes in asymptomatic offspring evoke high concerns [5–9]. Thus, animal models addressing sequelae in offspring may provide valuable information. In a pigtail macaque model, maternal ZIKV inoculation during gestation resulted in substantial brain lesions and silent brain pathology (i.e., periventricular T2-hyperintense foci and loss of fetal noncortical brain volume, injury to the ependymal epithelium with underlying gliosis, and loss of late fetal neuronal progenitor cells) in fetuses, even in the absence of microcephaly [10,11]. Two very recent studies in immunocompetent mouse models reported neurocognitive disorders and neurobehavioral deficits in offspring affected with mild congenital ZIKV infection [12,13]. These pioneering studies provided critical information regarding outcomes of mild congenital ZIKV infection in mouse offspring. Although, in these models, ZIKV induced clinical disease with reduced fetal birth weight, postnatal growth impediments, and neurobehavioral deficits. Thus, models reproducing subclinical *in utero* infection and long-term silent health sequelae (e.g., molecular pathology which is difficult to identify with diagnostic tests in clinical settings) in offspring in the absence of congenital Zika syndrome are not reported. While postnatal ZIKV infection in macaque infants resulted in altered emotional reactivity to acute stress [14], the evidence is still lacking for the cumulative impact of subclinical *in utero* ZIKV infection and postnatal environmental insults on health sequelae in offspring. This knowledge is important because secondary insults during postnatal life can unmask consequences of *in utero* acquired silent pathology [15,16].

Pigs are relevant to model human *in utero* ZIKV infection [17–19] and associated immunopathology and brain pathology in offspring because both species have similar physiology, genetics, immunity [20–27], fetal brain development and postnatal brain growth [28–31]. We and others have recently developed a fetal pig model which reproduces key aspects of *in utero* ZIKV infection in humans with persistent infection in the fetal brain, fetal membranes, and placenta [17–19]. Similarly to human and mouse infections [32,33], outcomes of infection in the porcine model depend on the gestational stage. Zika virus inoculation at the early stage of fetal development (25 gestation days, gd; the total duration of porcine gestation is 114 days) resulted in fetal death [19]. In contrast, fetuses infected at the mid-stage of development (50 gd) did not show brain lesions 28 and 60 days later [18,19].

Congenital ZIKV infection in mice increased *in utero* levels of type I IFNs [34,35], which was suggested to play a role in fetal demise [34]. In our porcine model studies, subclinical persistent *in utero* infection in mid-gestation also increased interferon alpha (IFN-α) levels in fetal blood plasma and amniotic fluid, while IFN-α was below the detection limit in all control fetuses [18]. In addition, levels of IFN-α positively correlated with ZIKV titers in fetuses. Interestingly, while fetuses did not have pathology or lesions, they showed persistent infection and dysregulation of more than 600 genes in their brains [18].

In the present study, to establish subclinical *in utero* infection, we exposed porcine litters to ZIKV at mid and late gestation, when similarly to humans, the fetal pig brain has a growth spurt [28,30,31,36]. We defined whether subclinical *in utero* infection imposes IFN-α sequelae and molecular brain pathology in offspring that did not have clinical signs of congenital Zika syndrome. We also tested whether prenatal exposure to subclinical ZIKV infection and postnatal social stress have a cumulative impact on immune responses and behavior in offspring.

## Results

### Exposure of fetuses to ZIKV at mid-gestation results in subclinical *in utero* infection with no clinical signs of congenital Zika syndrome in offspring

**Fig 1:**
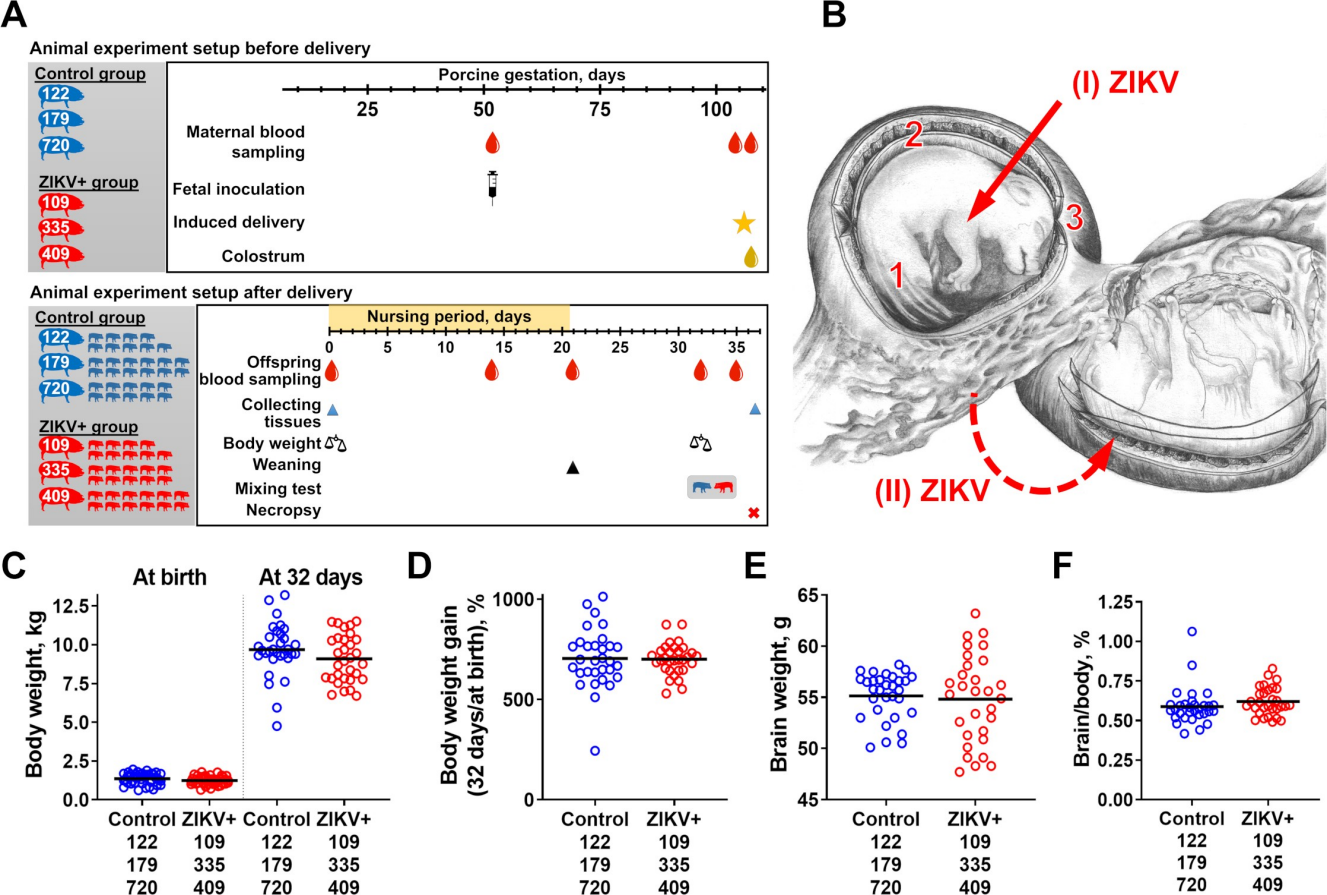
Animal experimental setup. **(A)** The actual number of experimental animals and sampling schedule for mothers and offspring. (**B**) A porcine uterus has multiple fetuses (on average pigs have 14–16 fetuses) with each fetus possessing individual amniotic membrane and placenta. Two fetuses in each pregnant pig were directly inoculated (**I**) with ZIKV. See **S1 Video** for ultrasound-guided inoculation. Afterward, ZIKV spreads (**II**) between siblings and causes productive infection in (**1**) amniotic membranes, (**2**) placenta, and (**3**) fetal brains of directly inoculated and trans-infected not-manipulated fetuses [17–19,37]. (**C**) Body weight, (**D**) body weight gain, (**E**) brain weight, and (**F**) brain/body weight ratio in control and ZIKV-exposed offspring. Solid lines represent mean values. Brains from offspring were collected at necropsy. See **S1A Table** for individual values.

To induce subclinical *in utero* infection, we directly inoculated two conceptuses (a fetus with fetal membranes; on average pigs have 14–16 fetuses) from three sows with 10^5^ TCID_50_/fetus of the ZIKV PRVABC59 strain at 53–54 gd (**S1 Video; Fig 1A and 1B; S1 Fig**). Litters with sham-inoculated conceptuses from three sows were used as controls.

**Fig 2:**
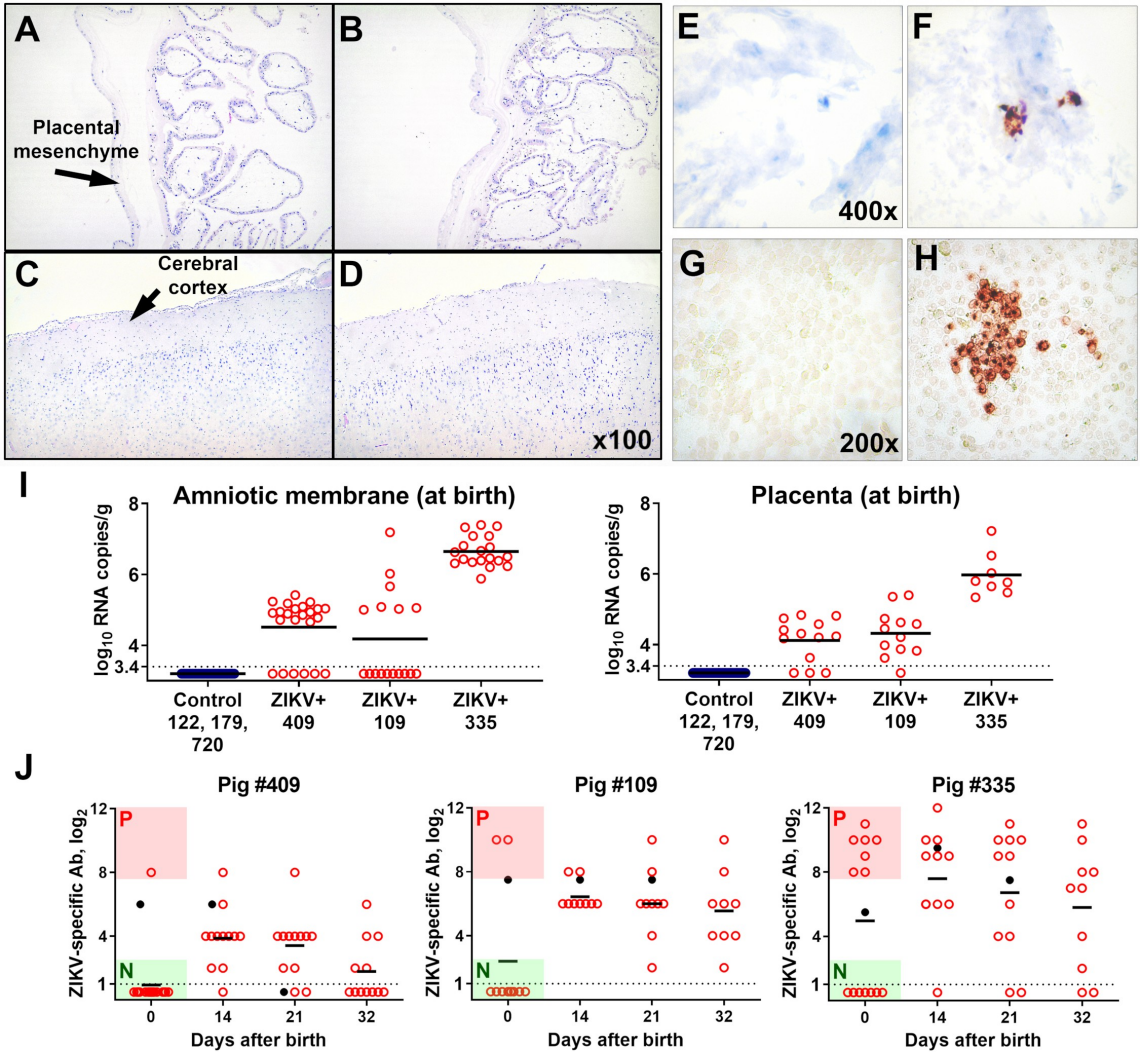
Histology, viral loads, and ZIKV-specific Ab responses. Hematoxylin-eosin staining in the placenta (sampled at birth) (**A**: control sow #122; **B**: ZIKV-inoculated sow #335) and neonatal brain (sampled at euthanasia, 37 days) (**C**: control piglet #2, sow 720; **D**: piglet #7 from ZIKV-inoculated sow 409). ZIKV-specific *in situ* hybridization in the placenta (sampled at birth) from control (**E**) and ZIKV (**F**) litters. Positive cells were found in a sample from pig #335. An immunoperoxidase monolayer assay (IPMA) to detect and quantify ZIKV-specific IgG Ab in porcine blood plasma. Blood plasma from control (**G)** and ZIKV-exposed (**H**) offspring (sampled at birth). (**I**) ZIKV RNA loads in amniotic membrane and placenta determined by RT-qPCR. Solid lines represent mean values. The dotted line represents the limit of detection (LOD). See **S1B Table** for individual values. ZIKV-specific Ab in offspring blood plasma detected by IPMA (**J**). All samples from the control litters were negative. Black dots–Ab titers in maternal blood. Offspring blood at birth was collected before first colostrum feeding. Offspring were subdivided into two subgroups based on ZIKV-specific serological status at birth: negative for Ab at birth–“N” and positive for Ab at birth–“P.” Solid lines represent mean values. The dotted line represents LOD. See **S1C Table** for individual values.

Mothers did not show clinical signs. All pregnant pigs were synchronized and delivered at term (114–115 days). Control and experimental litters contained 10.6% and 15.9% dead newborns, respectively (**S1A Table;** *P* = 0.67), which is in line with usual rates of fetal mortality in pigs [38,39]. The number of weak piglets was also similar in both groups (**S1A Table**, *P* = 0.85). *In utero* Zika virus exposure did not significantly affect cranium diameter in piglets (*P* = 0.41; **S1A Table**). While body weights at birth were lower in the ZIKV group (ZIKV group: 1.24±0.27 kg, control group: 1.36±0.35 kg, *P* = 0.05) (**Fig 1C**), body weight gain was not affected (*P* = 0.92) (**Fig 1C and 1D**). Brain weights in the ZIKV group (**Fig 1E**) had a slightly wider (*P* = 0.66) distribution (ZIKV group: coefficient of variation 7.9%, control group: coefficient of variation 4.2%). Brain to body weight ratio (**Fig 1F**) was also not affected (*P* = 0.08). Placental samples collected at birth and offspring brains did not have histopathological lesions (**Fig 2A–2D**).

To confirm *in utero* infection, we demonstrated high loads of ZIKV in amniotic membranes and placenta from all three ZIKV-exposed litters (**Fig 2I; S1B Table**). We also detected ZIKV RNA by *in situ* hybridization in the placenta and by RT-qPCR targeting the negative strand of ZIKV RNA in the placenta and amniotic membranes (**Fig 2E and 2F; S1B Table)**. These results confirm ZIKV transmission between siblings and productive, persistent infection in fetal membranes and placenta.

All samples from control mothers and piglets were negative for ZIKV-specific IgG antibodies (Abs) (**S1C Table**). *In utero* ZIKV exposure caused maternal infection as indicated by virus-specific IgG Ab in maternal plasma (**Fig 2J**; **S1D Table**). Most probably, maternal infection was transient because maternal blood plasma samples were free for ZIKV RNA at all five sampling time-points as determined by RT-qPCR (**S1B Table**). Moreover, in our previous studies, where we induced more severe *in utero* infection with higher viral doses, maternal endometrium and lymph nodes were free from ZIKV [18,19,37]. Next, we determined whether ZIKV replicated in fetuses and persisted in offspring. ZIKV-specific IgG Abs were detected in blood plasma from a subset of newborns in all three exposed litters (**Fig 2J; S1C Table**). In pigs, maternal Abs do not pass to porcine fetuses through the placenta [40]; however, it is being transferred passively to offspring via colostrum. Blood plasma samples from all newborns were collected before first colostrum ingestion, and high Ab titers demonstrated productive fetal infection and subsequent *in utero* Ab responses (**Fig 2J; S1C Table**). Two ZIKV-specific RT-qPCR assays did not show viral RNA in the blood plasma, cerebrum, and cerebellum from all exposed and control piglets (including stillborn and weak piglets) (**S1B Table**).

Altogether, we induced subclinical persistent *in utero* infection which did not cause readily identifiable clinical pathology and productive infection in offspring.

### Subclinical *in utero* ZIKV infection is associated with IFN-α sequelae in affected offspring

**Fig 3:**
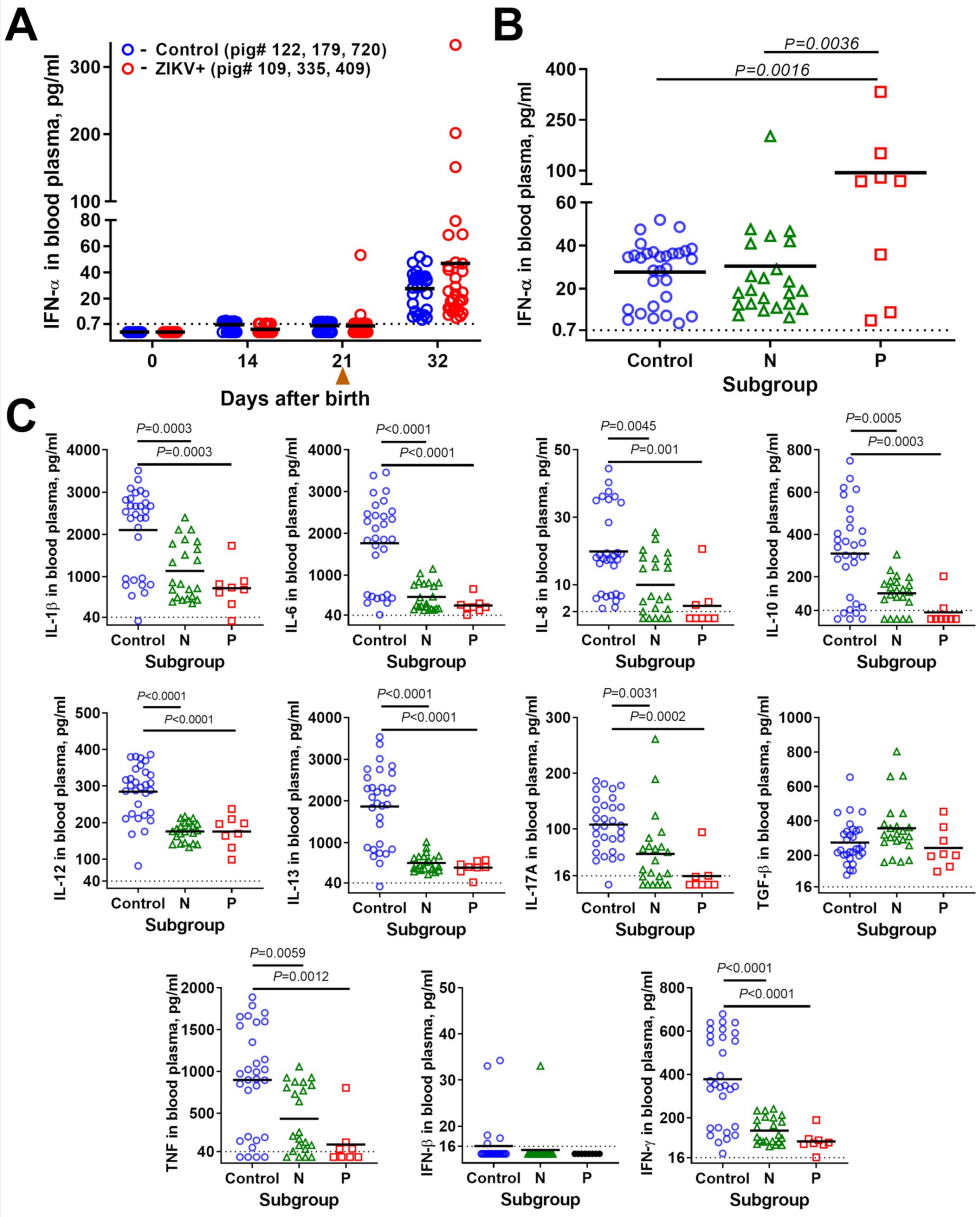
Cytokine levels in offspring blood plasma. (**A**) Kinetics of IFN-α in offspring blood plasma. An arrowhead (▲)–weaning at 21 days after birth. (**B**) IFN-α in offspring blood plasma at 32 days after birth. Solid lines represent means. The dotted line represents the limit of quantification (LOQ). See raw data in **S1C Table** for individual values. (**C**) Cytokine levels in offspring blood plasma at 32 days after birth. See raw data in **S1E Table** for individual values. Offspring subgroups: **N**–negative for endogenous ZIKV-specific Ab at birth; **P**–positive for endogenous ZIKV-specific Ab at birth.

Subclinical ZIKV infection in porcine conceptuses increases concentrations of IFN-α in amniotic fluids and fetal blood at 28 days after inoculation [18,37]. Here, we defined whether subclinical *in utero* infection imposes IFN-α sequelae in affected offspring. Within 21 days after birth, IFN-α (**Fig 3A**) as well as IL-1*β*, IL-6, IL-8, IL-10, IL-12, IL-13, IL-17A, TGF-*β*, TNF, IFN-*β*, and IFN-γ levels (at birth) remained below or at the detection limit in control and ZIKV-exposed piglets. The same levels in both offspring groups suggest that maternal IFN-α (which could be transferred with milk within the first 21 days of life) equally affected offspring in both groups during the nursing period. Two ZIKV piglets, however, showed an increase in IFN-α levels already at 21 days (at weaning–separation of mother from offspring) (**Fig 3A**). While piglets from both control and ZIKV litters had detectable IFN-α levels at 32 days, 19% of ZIKV piglets showed considerably increased levels of IFN-α in their blood plasma (**Fig 3A; S1C Table**). Next, we analyzed IFN-α responses in offspring subdivided into two subgroups based on ZIKV-specific serological status at birth: negative for Ab at birth–“N” and positive for Ab at birth–“P,” (**Fig 2J**; **S1C Table**). Remarkably, the increased IFN-α levels were mostly attributed to the P subgroup with high ZIKV-specific Ab titers at birth (**Fig 3B**). IFN-α levels in the P subgroup were significantly higher than in the control group and N subgroup, indicating that IFN-α increase may correlate with the serological status in offspring at birth. Maternal IFN-α likely did not affect increased IFN-α responses in ZIKV offspring at 32 days because maternal blood IFN-α levels in ZIKV litters were lower or equal to that in control litters (*P* ≥ 0.23) (the same was observed in colostrum, *P* = 0.4; **S1D Table**) and did not change significantly throughout the study (**S2 Fig**). Moreover, the IFN-α increase in offspring was detected at 32 days, eleven days after separation of piglets from mothers. In addition, maternal IFN-α is unlikely to affect fetuses and offspring as it does not cross through the human [41] or porcine placenta [18,42]. All of other eleven tested cytokines did not show the increase (**Fig 3C; S1E Table)**, suggesting specific IFN-α pathology.

We do not know whether lower levels of IL-1*β*, IL-6, IL-8, IL-10, IL-12, IL-13, IL-17A, TNF, and IFN-γ in ZIKV offspring at 32 days (**Fig 3C; S1E Table**) were caused by subclinical *in utero* ZIKV infection or maternal cytokine background (**S2 Fig**). Maternal blood levels of these cytokines were lower in the ZIKV group (although the difference was not statistically significant) (**S2 Fig**) that could potentially contribute to the lower cytokine levels in offspring. Previously described markers of maternal immune activation which may affect fetal health, IL-6 [43] and IL-17A [44] in the ZIKV group were lower or equal to that in control litters (*P* ≥ 0.47); these data are in agreement with findings in pregnant women with acute ZIKV infection [45] and mice [12]. Also, IL-6 and IL-17A levels in ZIKV group did not change significantly throughout the experiment (**S2 Fig**); the same was found for maternal IL-1*β*, IL-8, IL-10, IL-12, IL-13, IFN-*β*, and IFN-γ (**S2 Fig**).

Overall, these data suggest that subclinical *in utero* ZIKV infection, without active maternal infection and changes in maternal cytokines, may specifically affect IFN-α response in offspring.

### Combined exposure to subclinical prenatal ZIKV infection and postnatal social stress induces a synergistic pathological effect on IFN-α responses in affected offspring

We performed a mixing test (**S2 Video**) on control and affected piglets at 35 days of age to identify whether subclinical *in utero* ZIKV infection and social stress have synergistic effects on cytokine responses in offspring.

**Fig 4:**
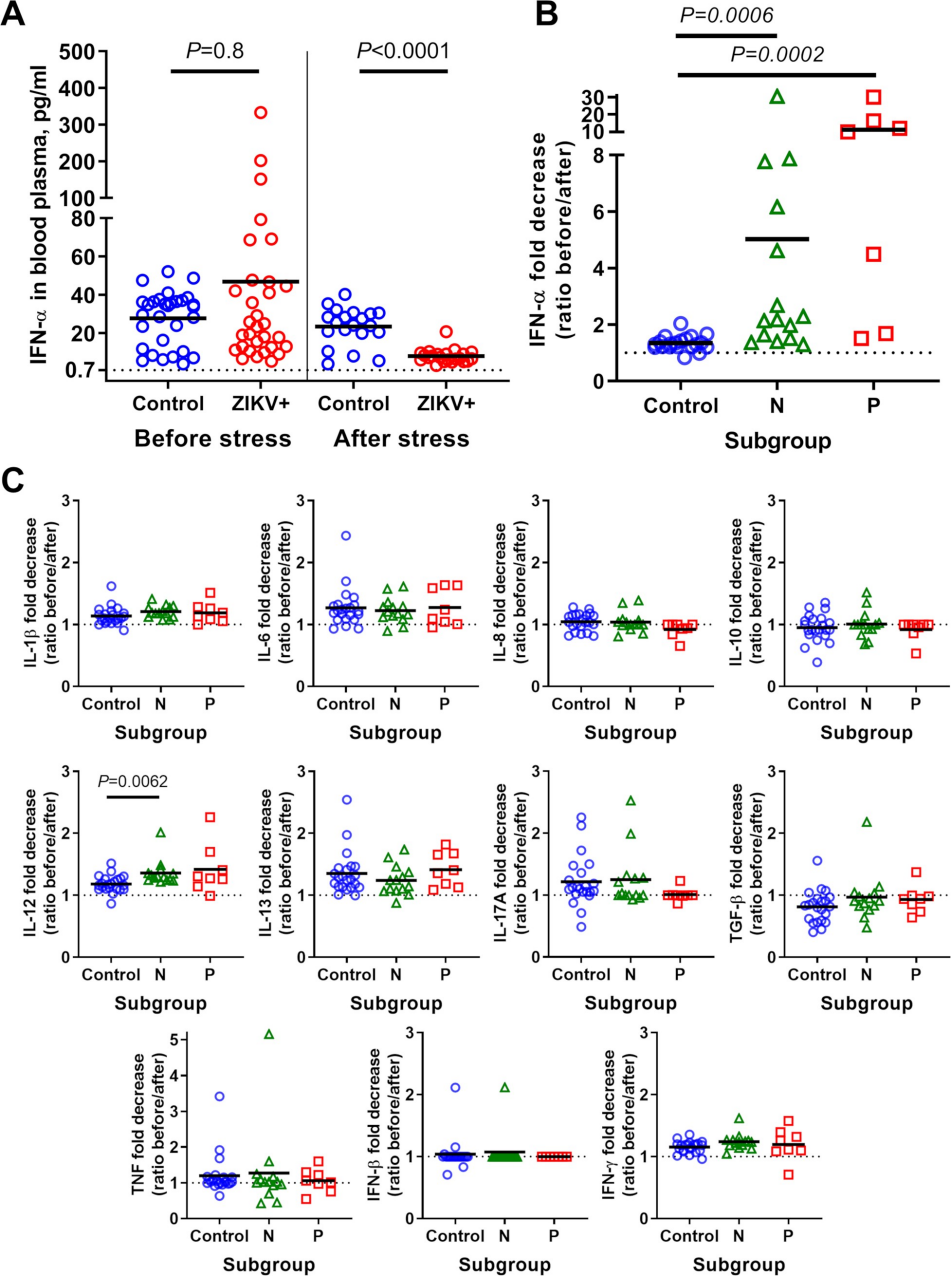
IFN-α shutdown in offspring after social stress. (**A**) IFN-α in offspring blood plasma before and after social stress (the mixing test). (**B**) Fold decrease of IFN-α level after the mixing test. **(C)** Fold decrease of other tested cytokines after the mixing test. Offspring subgroups: **N**–negative for endogenous ZIKV-specific Ab at birth; **P**–positive for endogenous ZIKV-specific Ab at birth. The dotted line represents LOQ. For negative samples (below LOQ), fold change was calculated using LOQ value as a baseline. Solid lines represent means. See raw data in **S1C** and **S1E Table** for individual values.

After the mixing test, piglets in the control group showed a slight, up to a 2-fold decrease (1.3±0.3) in blood plasma IFN-α levels in comparison to the levels before the mixing test (**Fig 4A; S1C Table**). In contrast, piglets affected with subclinical *in utero* infection showed dramatic, up to a 31-fold decrease (6.8±8.5) in blood plasma IFN-α levels (**Fig 4A**). This abrupt decrease in peripheral IFN-α was demonstrated by a considerable proportion of ZIKV-affected offspring and did not depend on the initial IFN-α level before stress induction. For example, ZIKV-affected offspring with exceptionally high IFN-α levels before mixing test showed similar or even lower IFN-α levels as in other groupmates after the mixing test (**S1C Table**, sow #409, piglet #7; sow #109, piglets #8 and #10; sow #335, piglets #2 and #12). Also, the decrease was observed not only in offspring with increased IFN-α levels. ZIKV-affected piglets which initially had IFN-α levels comparable to control piglets also showed a dramatic decrease after the mixing test (**S1C Table:** sow #409, piglet #13; sow #109, piglet #5; sow #335, piglets #5, #8 and #10). Next, we analyzed IFN-α responses in offspring subdivided into two subgroups based on ZIKV-specific serological status at birth (**Fig 4B; S1C Table**). ZIKV offspring in both P and N subgroups showed a statistically significant decrease in blood plasma IFN-α levels (**Fig 4B**). In sharp contrast, stress did not induce a significant decrease in other tested cytokines (0.96–1.38 mean fold change, **Fig 4C; S1E Table**). No difference in IFN-α shutdown was observed between serological (N and P; *P* = 0.12) and sex (*P =* 0.29) subgroups.

Our findings reveal synergistic interactions between subclinical *in utero* ZIKV infection and postnatal stress in promoting pathological IFN-α responses in affected offspring.

### Subclinical *in utero* ZIKV infection is associated with sex-specific molecular brain pathology in affected offspring

**Fig 5:**
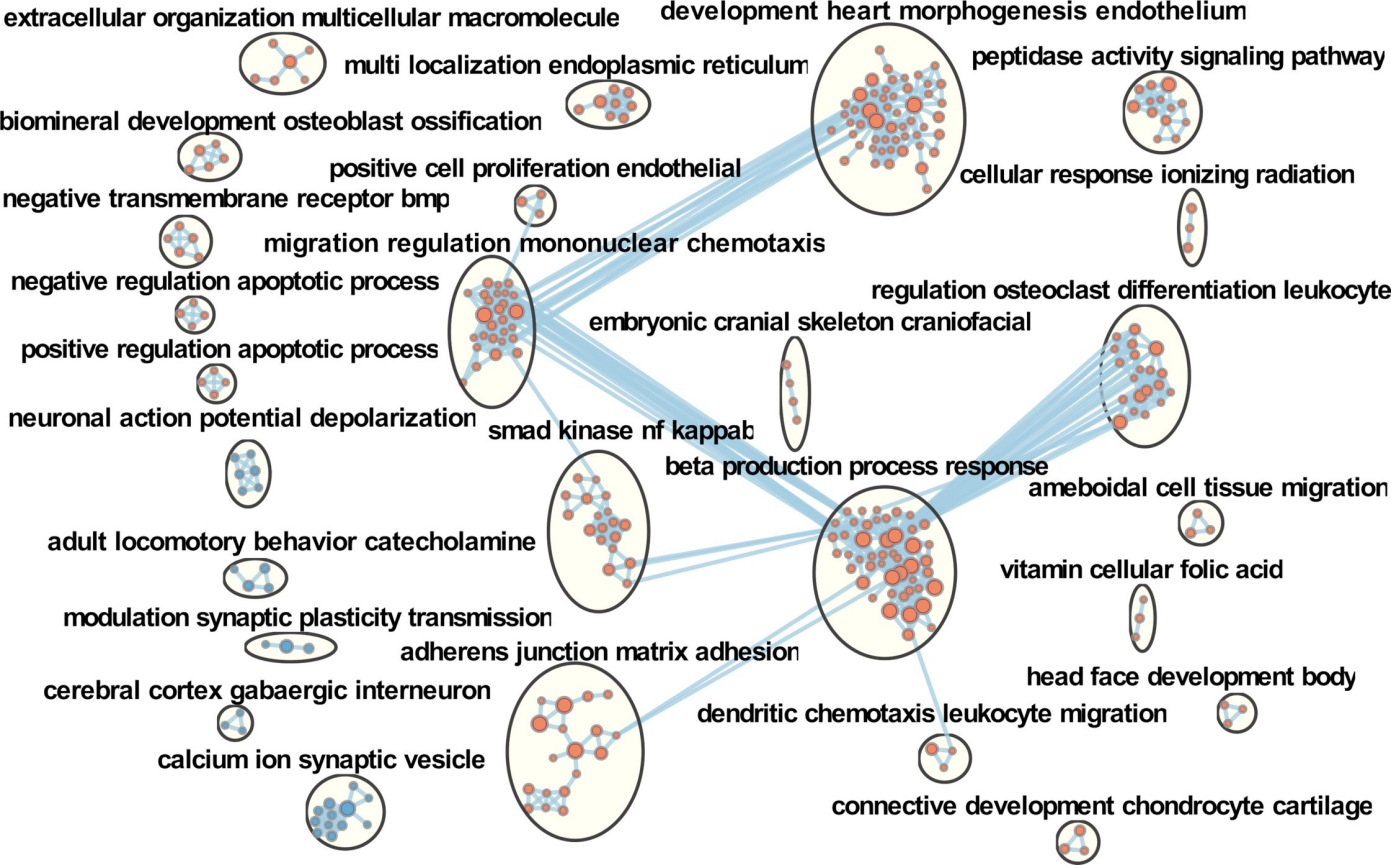
Transcriptional changes in the prefrontal cortex of offspring affected with subclinical *in utero* ZIKV infection (all Control offspring versus all ZIKV offspring). Molecular pathology network in the prefrontal cortex of offspring affected with subclinical *in utero* ZIKV infection. Enrichment map of significantly altered GO biological processes. Red are pathways with positive and blue are with negative enrichment. All subnetworks with FDR-adjusted *P* < 0.1 and at least three connected nodes are shown. See raw data in **S2B Table** for individual GO biological processes.

**Fig 6:**
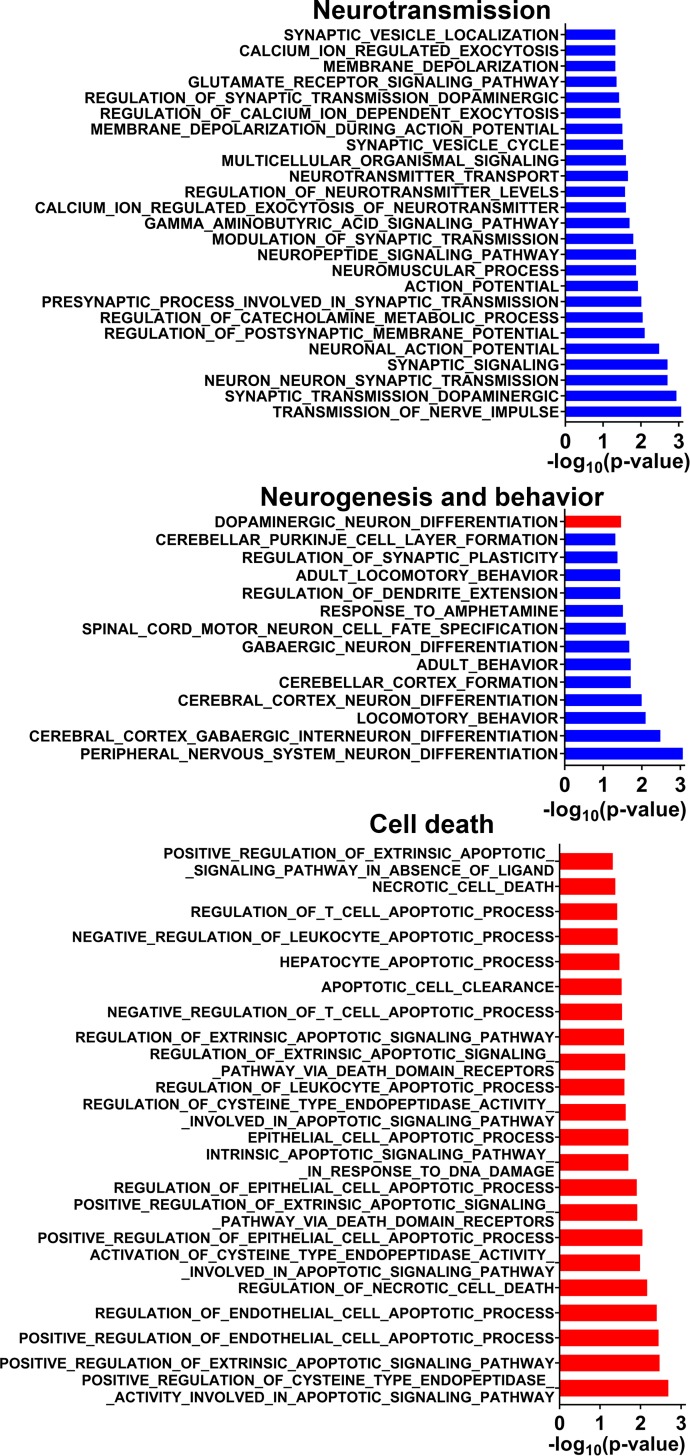
Neuronal and cell death biological processes in the prefrontal cortex of offspring affected with subclinical *in utero* ZIKV infection. FDR-adjusted *P* < 0.05. Blue bars–downregulated processes. Red bars–upregulated processes. See raw data in **S2B Table** for individual values.

We sought to characterize whole genome expression in the prefrontal cortex (PFC) of clinically normal offspring affected by subclinical *in utero* ZIKV infection. Brains from 30 ZIKV-affected offspring and 12 control offspring with no history of *in utero* infection were sampled at 37 days after birth and analyzed using RNA-seq (**S1C Table**). On a global transcriptional level, gene expression differed considerably between PFC samples from ZIKV-affected and control offspring (**S3A and S3B Fig**; **S2A Table**). Functional set enrichment of Gene Ontology (GO) biological processes also showed significant effects in brains of ZIKV offspring (**Fig 5; S2B Table**). Specifically, genes with altered expression in the PFC of virus-affected offspring were positively enriched for processes related to cell death (29 GO pathways related to apoptosis and necrosis; false discovery rate (FDR)-adjusted *P* < 0.1, **S2B Table**), cytokine responses and immunity (61 GO pathways; FDR-adjusted *P* < 0.1, **S2B Table**) and organ/tissue morphogenesis, development, and regeneration (130 GO pathways; FDR-adjusted *P* < 0.1, **S2B Table**). Whereas a large set of biological processes involved in neuronal function, i.e., synaptic transmission, GABAergic signaling, calcium ion regulation, cerebral cortex neuron differentiation, and others, were negatively enriched (64 GO pathways; FDR-adjusted *P* < 0.1, **S2B Table**) (**Fig 5**). More stringent analyses of GO biological pathways related to neuronal and cell death pathways with FDR-adjusted *P* < 0.05 is represented in **Fig 6**. Interestingly, “response to type I interferon” (FDR-adjusted *P* = 0.0026), “positive regulation of type I interferon production” (FDR-adjusted *P* = 0.026), “regulation of type I interferon production” (FDR-adjusted *P* = 0.011) and “response to interferon beta” (FDR-adjusted *P* = 0.08) GO processes were positively enriched in the PFC of affected offspring (**S3C Fig; S2B Table**). Another upregulated biological process was “response to corticosteroid” (FDR-adjusted *P* = 0.03) (**S3D Fig; S2B Table**), which is in line with previously reported dysregulation in genes related to physiological stress responses in porcine fetuses [18]. In support of the observed altered transcriptional profile of corticosteroid-responsive genes, fetuses showed significantly elevated *in utero* cortisol levels during persistent ZIKV infection [19]. To find out whether transcriptional and hormonal *in utero* cortisol disbalance associated with subclinical infection imposes sequelae in offspring, we measured cortisol concentrations in hair, a well-established test to assess chronic stress throughout the lifespan [46]. The cortisol levels were higher in ZIKV piglets (**S3E Fig; S1F Table**) providing considerable support of transcriptional findings in affected offspring. A previous study in rhesus macaques demonstrated that even moderate increase in mean hair cortisol levels (1.59 times) is indicative of chronic stress [47]. The difference in porcine offspring, however, was not statistically significant (mean—1.27 times; *P =* 0.17). Thus, the relation between subclinical *in utero* ZIKV infection and chronic stress in offspring remains to be further confirmed.

Next, we generated gene sets compiled from MalaCards (http://www.malacards.org) and previous publications [18] (**S2C Table**) linked to the following clinical disorders associated with congenital Zika syndrome in human fetuses and neonates: microcephaly, epilepsy, dysphagia, clubfoot, and arthrogryposis. Additionally, we collected gene sets from MalaCards for Guillain-Barré Syndrome, schizophrenia, attention deficit-hyperactivity disorder, psychotic disorder, anxiety disorder, mood disorder, and learning disability (**S2C Table**) [18]. Like in the previous fetal RNA-seq study [18], Gene Set Enrichment Analysis (GSEA) showed that ZIKV-affected offspring were negatively enriched in the schizophrenia gene set (FDR-adjusted *P* = 0.001) (**S2D and S2E Table**).

**Fig 7:**
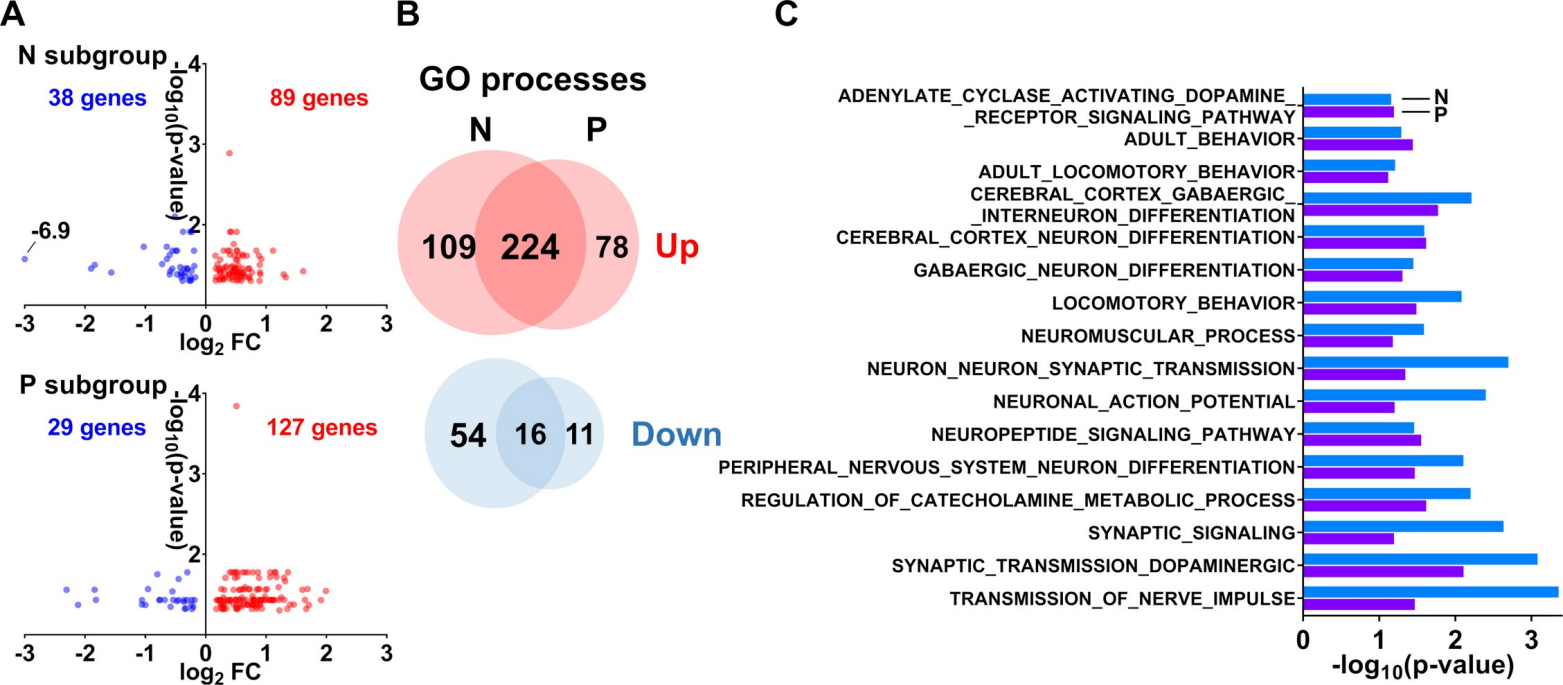
Molecular pathology in the prefrontal cortex of ZIKV offspring from N and P subgroups. **(A)** Volcano plots of the upregulated (red) and downregulated (blue) genes in N and P subgroups. FDR-adjusted *P* < 0.05. See raw data in **S2F** and **S2G Table** for individual gene values. (**B**) Venn diagram of individual and shared GO processes in the N and P subgroups. FDR-adjusted *P* < 0.1. See raw data in **S2H–S2J Table** for individual process values. (**C**) Bar plot of shared neuronal and behavioral GO processes in the N and P subgroups. FDR-adjusted *P* < 0.1. See raw data in **S2J Table** for individual process values.

To determine whether offspring with the distinct ZIKV-specific serological status at birth have molecular pathology in the brain, we compared RNA-seq data between N and P subgroups. Offspring in both subgroups showed a similar number of downregulated and upregulated genes (**Fig 7A; S2F and S2G Table**). A considerable number of upregulated and downregulated GO processes (**Fig 7B; S2H–S2J Table**), including neuronal and behavioral processes (**Fig 7C; S2J Table**), were also shared between N and P subgroups, which is likely reflective of the similar gene expression profiles. Collectively, this analysis suggests that subclinical *in utero* ZIKV infection may cause similar molecular pathology in the brain of offspring both positive and negative for ZIKV-specific Ab at birth.

**Fig 8:**
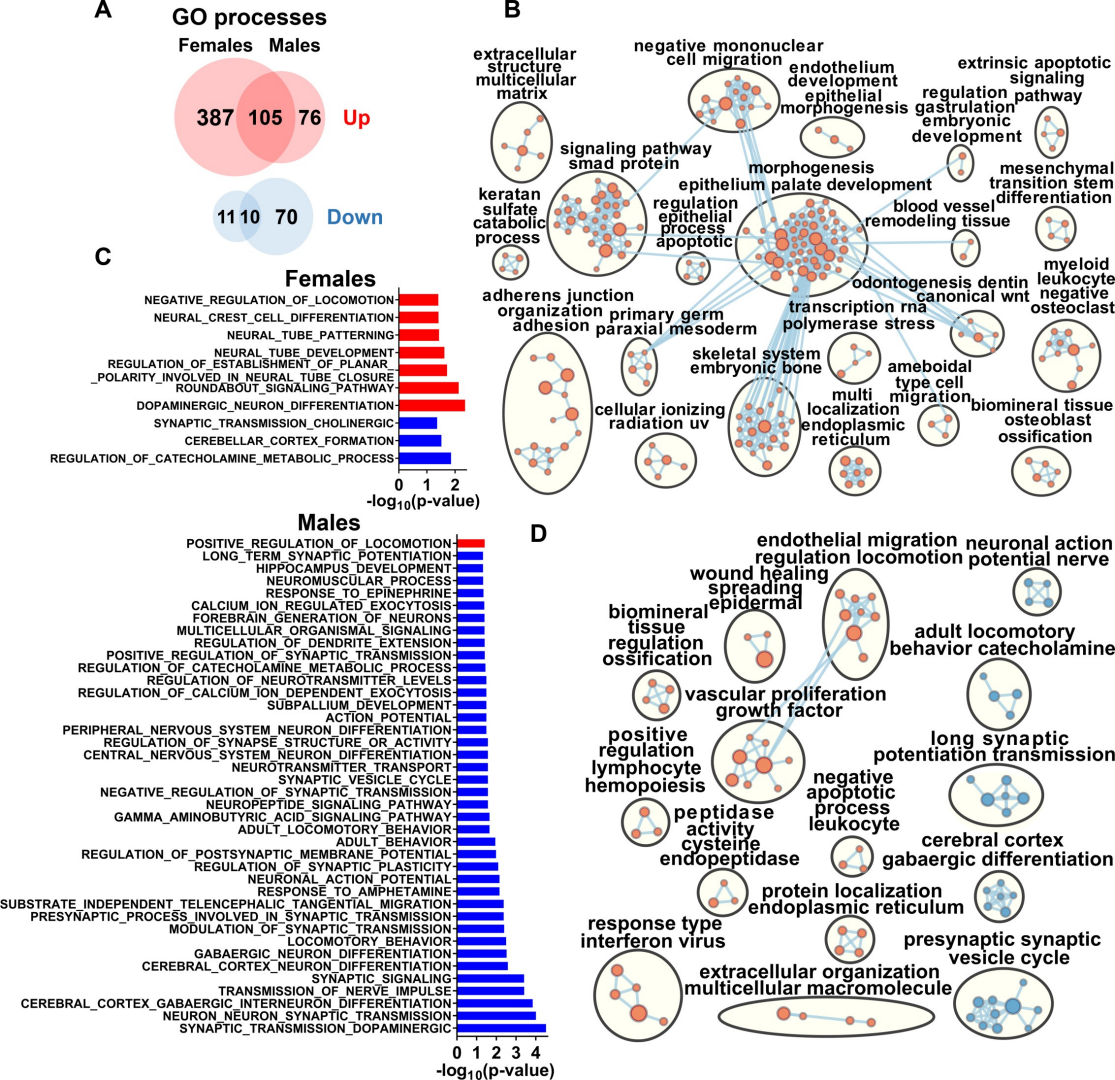
Sex-specific molecular pathology in the prefrontal cortex of offspring affected with subclinical *in utero* ZIKV infection. (**A**) Venn diagram of individual and shared GO processes in the female and male subgroups. FDR adjusted P < 0.1. See raw data in **S2K–S2M Tables** for individual process values. Enrichment map of significantly altered GO biological processes in the prefrontal cortex of female (**B**) and male (**D**) offspring affected with subclinical *in utero* ZIKV infection. Pathways with positive (red) and negative (blue) enrichment are shown. All subnetworks with FDR-adjusted *P* < 0.1 and at least three connected nodes are shown. (**C**) Positive (red) and negative (blue) enrichment of neuronal and behavioral GO processes in female and male subgroups. FDR adjusted *P* < 0.05.

Next, we analyzed molecular pathology in female and male offspring affected with subclinical *in utero* ZIKV infection. Female and male offspring showed a distinct transcriptional signature in the PFC (**Fig 8A**; **S2K and S2L Table**). The considerably larger number of upregulated pathways in female offspring represented by developmental, differentiation, morphogenesis, cell migration, transcription, catabolic, cell adhesion/junction, signaling, and tissue remodeling processes (**Fig 8B**), may signify more intensive compensatory responses to sequelae of *in utero* infection. While females and males shared 118 enriched biological processes (**S2M Table**), considerably larger loss of affected neuronal and behavior pathways (FDR-adjusted *P* < 0.05) was observed in males (**Fig 8C**). Enrichment analysis of significantly altered processes also attributed neuronal and behavioral pathways to male offspring (**Fig 8B** and **Fig 8D**). This suggests a greater loss of neuronal function in males, which is in a strong agreement with more prominent ZIKV-induced neurocognitive pathology in male mouse offspring [12].

**Fig 9:**
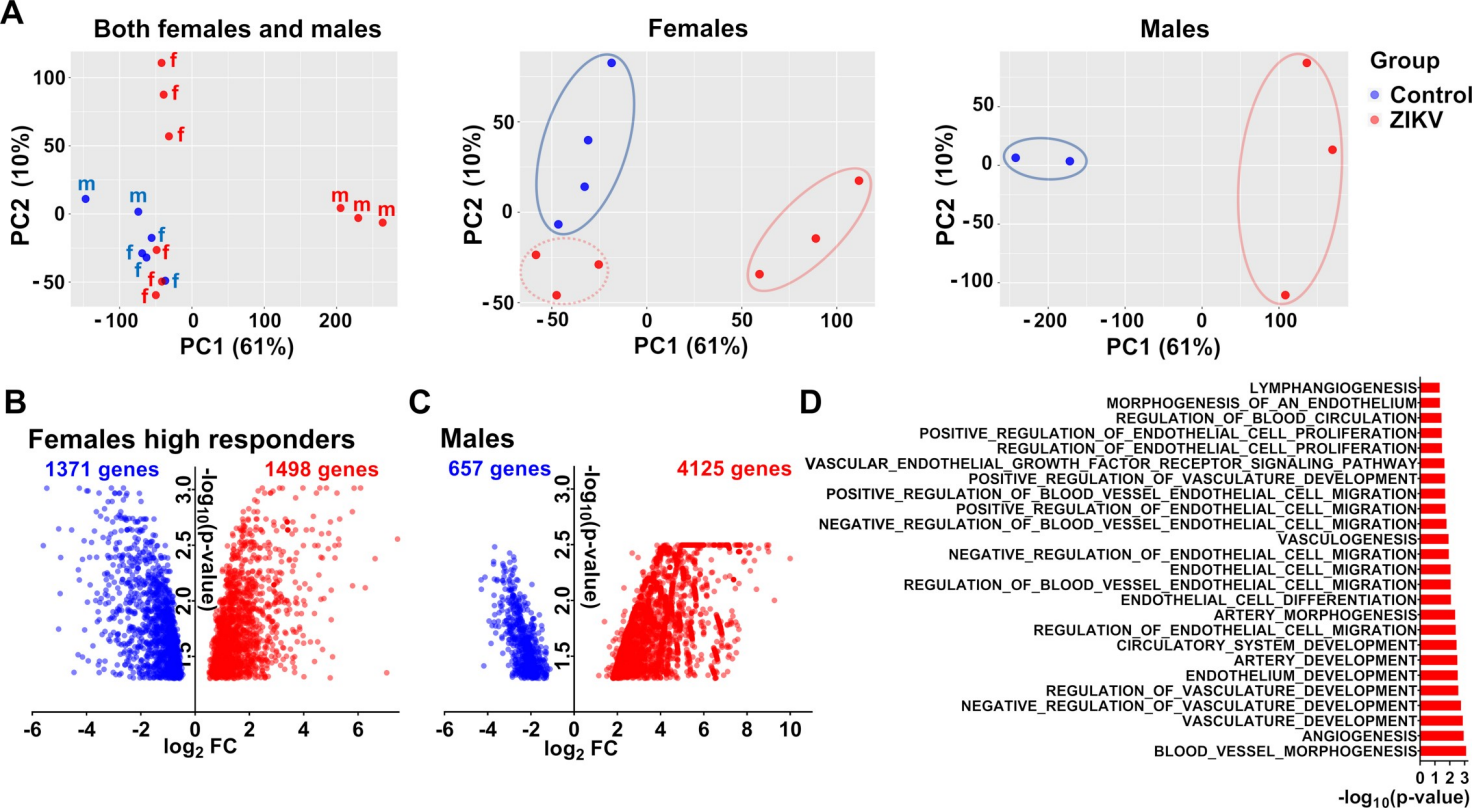
Sex-specific changes in the global transcriptional signature in the placenta with persistent ZIKV infection. **(A)** Principal-component analysis (PCA) of RNA-seq data in the placenta with persistent ZIKV infection. Female low-responders are in the left low corner. Volcano plots display significantly affected genes (*P* < 0.05) in female (**B**) and male (**C**) placental samples. Blue and red dots indicate downregulated and upregulated genes, respectively. (**D**) A sample size of male control placentae (**Fig 9A**) motivated us to focus on GSEA analysis in only placental samples of female high responders (**S2Q Table**). GO biological vascular processes significantly altered (FDR-adjusted *P* < 0.05) in the placenta with persistent ZIKV infection. See raw data in **S2Q Table** for individual GO biological processes.

Although humans and pigs have different placentation types (hemochorial and epitheliochorial, respectively) that prevents studies on mechanisms of virus and Ab transmission from mother to fetus, a fetal placental mesenchyme in pigs has all major cell types found in the human fetal side of the placenta (chorionic plate) and performs the same fundamental functions [48–50]. Similar to replication in the human placenta [51], ZIKV replicates in the porcine fetal placental mesenchyme [18,37]. Also, we have recently demonstrated that similar to human placental infection [51], ZIKV infection in the porcine placental mesenchyme is associated with the increased number of CD163-positive cells [37]. Thus, to provide independent support for the sex-specific pattern of transcriptional responses to ZIKV infection, we profiled the whole genome expression in placental samples persistently infected with ZIKV (**S1B Table**). We observed that on a global transcriptional level, gene expression signature in the infected fetal placental samples also exhibited the considerable sex-specific pattern (**Fig 9A; S2N–S2P Table**). After correcting gene expression data for sex the principal component analysis showed that while female and male control samples were grouped close to each other, ZIKV-infected samples had a strong sex-specific separation indicating a strong dependency of ZIKV-induced transcriptional changes on animal sex. Zika virus infection may cause discordant clinical outcomes in human dizygotic twins, ranging from severe disease to asymptomatic infection, and different whole-genome transcriptional responses in neuronal progenitor cells of twins [52,53]. In accordance, among females, we found three low-responders which grouped very closely with control samples (**Fig 9A; S2N Table**). Other three samples were high-responders (**Fig 9A; S2O Table**). Non-responder female samples were from the same litter (#109). Interestingly, both low- and high-responder subsets had the high placental viral loads (low-responders: 4.74–5.40 log_10_ ZIKV RNA copies/g, high-responders: 4.74–6.02 log_10_ ZIKV RNA copies/g**; S1B Table)**. The number of differentially expressed genes in ZIKV-positive placental samples was considerably higher than in ZIKV-positive brain samples, which is in agreement with ongoing productive placental infection (**Fig 2I**; **S1B Table**). The high number of altered genes in both female and male samples is also in agreement with *in vitro* RNA-seq studies in placental cells [54] and *in vivo* studies in human cells [55]. The higher number of affected genes in samples from males (**Fig 9B and 9C**; **S2O and S2P Table**) might suggest a stronger potential for pathological outcomes in males than in females [12]. A large set of affected biological processes (FDR-adjusted *P* < 0.05) represented blood vessel and endothelial development, proliferation, migration, and morphogenesis (**Fig 9D, S2Q Table**). This is in agreement with studies in mice [56] and rhesus macaques [57] where ZIKV-induced vascular pathology was described. Additionally, biological pathways related to actin, extracellular matrix, and syncytium formation were enriched (**S2Q Table**).

Collectively, RNA-seq results in the brain of affected offspring and virus-infected placental samples, which both did not show histopathology and lesions, provide strong evidence for silent sex-specific molecular pathology.

**Fig 10:**
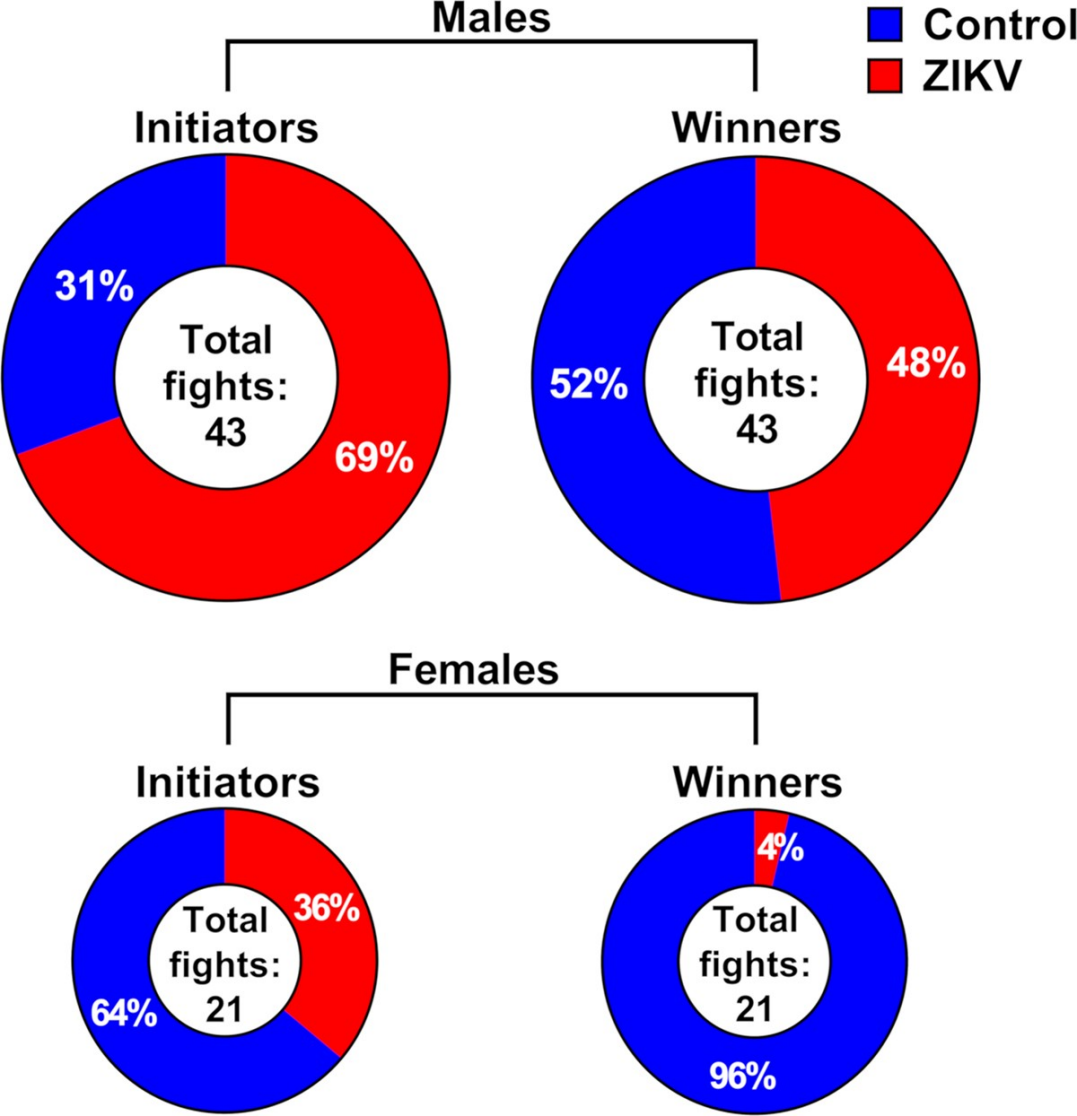
Behavioral stress responses in offspring. Mixing tests were performed between control and ZIKV-affected offspring to induce social stress and assess behavior. See raw behavioral data in **S1G Table**.

Next, we tested whether molecular pathology in the brain of affected offspring is associated with altered behavior in the normal environment and during stress. We did not observe differences between animal groups in the regular environment, in a pen with mother and littermates (20 days after birth—38.2±4.4% of active control piglets; 38.5±1.1% of active ZIKV piglets), after maternal removal (21 days after birth—25.5±4.7% of active control piglets; 29.6±3.9% of active ZIKV piglets), or one day after maternal separation (22 days after birth—25.8±3.3% of active control piglets; 25.5±0.5% of active ZIKV piglets). Then, we tested responses under stressful conditions using the mixing test. Mixing unfamiliar piglets often results in aggressive fighting to establish a dominance hierarchy [58]. Using this behavioral pattern, we compared fighting in control and ZIKV offspring (**S2 Video**). The percentage of initiated and won fights (individually for males and females) were compared against the expected value (50% versus 50%) in control versus ZIKV groups. The percentage of fights initiated by ZIKV male piglets (mean 69.4%) was more than twice higher than the percentage of fights initiated by control male piglets (mean 30.6%) (*P =* 0.13). (**Fig 10; S1G Table**). Won fights in males were evenly distributed between control and ZIKV groups (51.8% versus 48.2%; *P =* 0.92). In contrast, control females demonstrated more aggressive behavior than ZIKV females as represented by 63.9% of initiated fights (*P =* 0.45) and 96.3% won fights (*P* = 0.0039) (**Fig 10; S1G Table**).

Altogether, the lack of behavioral differences between control and affected groups in the regular environment and distinct behavioral patterns between groups during the mixing test suggest that subclinical *in utero* ZIKV infection and altered gene expression in the PFC might affect behavior in offspring in the stressful environment.

## Discussion

We addressed a question of whether subclinical *in utero* ZIKV infection may pose health sequelae in offspring in the absence of congenital Zika syndrome. There are two key findings from this study. First, subclinical *in utero* ZIKV infection was associated with abnormal IFN-α responses in apparently healthy offspring under normal environmental conditions and during social stress. Second, offspring affected with subclinical *in utero* infection showed the profoundly altered transcriptional signature in the brain free for ZIKV and lesions.

Here, like in previous mouse [35,59] and non-human primate ZIKV studies [60], we used *in utero* inoculation because maternal inoculation in pigs does not cause fetal infection [17]. However, the present study in offspring clearly demonstrates that subclinical, relatively isolated, ZIKV infection of the fetal-placental unit, without active maternal infection and changes in maternal cytokines, may cause long-term silent immunopathology and brain molecular pathology in offspring. This is the important finding suggesting that in addition to well-recognized maternal immune activation [61], rationale combinatory therapeutic interventions against congenital infections and long-term sequelae should also target specific fetal immunopathology. Among others, ZIKV-agitated *in utero* IFN-α responses of the fetal and placental origin [18,34,35] can be considered as therapeutic targets.

Based on our previous fetal studies and present findings in offspring, we suggest a link between the fetal IFN-α pathways reprogrammed during *in utero* ZIKV infection [18,37] and altered IFN-α responses in offspring (**Figs 3 and 4**). The hypothesis of fetal origins of adult disease was first described by David Barker, who proposed that disruptions to the *in utero* environment during fetal development program increase risks for disease during adulthood [62]. Subsequently, many studies confirmed that adverse effects during fetal development may program postnatal immune and neurological pathology which can be transferred across generations [63–66]. Our data also suggest age-specific IFN-α responses in the porcine model of subclinical *in utero* ZIKV infection. During fetal development, IFN-α levels in fetal blood and amniotic fluids are increased for several weeks after *in utero* inoculation [18], with a subsequent drop to undetectable levels at around 60 days after inoculation (**S4 Fig**). Afterward, offspring show elevated levels of IFN-α at around 21–32 days after birth (**S4 Fig**). In support of our findings, the age-specific nature of *in utero* acquired neurodevelopmental, behavioral, cognitive, and neuroimmune defects has been experimentally demonstrated [16,36,67–71]. For example, immune activation during mouse pregnancy causes transiently elevated brain cytokine levels in offspring at birth, decreased levels postnatally, and then elevated levels during adulthood [67]. Presumably, maturation of the immune and endocrine systems is required to trigger prenatally acquired neurodevelopmental pathology in offspring [72–74]. Factors determining the age-specific nature of the *in utero* acquired ZIKV-induced systemic immunopathology remain to be defined. While cerebrum and cerebellum were negative for ZIKV, we cannot fully exclude persistent infection in offspring because lymphoid tissues were not tested; however, in our previous study newborn piglets inoculated with 10^5.8^ TCID_50_ ZIKV intracerebrally, intradermally, or intraperitoneally cleared the virus from lymphoid tissues within seven days [75]. Additional studies, are required for comprehensive testing of multiple organs for persistent ZIKV infection in offspring affected with subclinical *in utero* infection and its connection with altered IFN-α responses.

A secondary insult during postnatal life may be necessary to unmask the silent consequences of *in utero* immune activation [15,16]. Here, we used the mixing test, a validated approach to model social confrontation in pigs [76–79]. Mixing a pair of unfamiliar individuals in a new environment induces stress, to which animals respond with physiological changes. The test induces cumulative stress from the separation of littermates, confrontation with a novel environment (a mixing chamber), confrontation with the unfamiliar conspecific and aggression (fight)-induced stress (**S2 Video**). Possible effects of sex, body weight, and familiarity with the environment, which may influence aggressive/social behavior and outcomes of the test [76], were excluded by mixing piglets of the same sex and similar weights in unfamiliar mixing chambers. We demonstrated that combined exposure to subclinical prenatal ZIKV infection and postnatal social stress induces synergistic pathological effect promoting abrupt IFN-α shutdown in affected offspring. Further attempts to better understand silent pathology and develop alleviative interventions in ZIKV-affected offspring should take into account synergistic interactions of multiple environmental insults.

The IFN-α increase and IFN-α shutdown apparently have different, yet unknown, mechanisms as indicated by different responses between N and P subgroups in the normal environment (**Fig 3B**) and during stress (**Fig 4B**). Abrupt nature of both phenomena, however, is most probably attributed to changes in peripheral blood cells. Because blood monocytes and dendritic cells are the primary sources of IFN-α in humans [80,81] and pigs [82], these cells should be studied in terms of *in utero* acquired ZIKV-induced IFN-α pathology. We also suggest studying hematopoietic stem cells in affected fetuses and offspring since excessive IFN-α may impact proliferation and reconstituting ability in hematopoietic stem cells and stem cell niche [83–85]. Interestingly, in contrast to the altered molecular signature in the brain and placenta, the increase of IFN-α in porcine offspring blood did not have the sex-specific nature, suggesting that brain/placenta-specific phenomenon and described IFN-α immunopathology in blood most probably have different mechanistic background.

To our knowledge, type I IFN profiles in human fetuses and offspring affected with ZIKV infection were not addressed, yet. However, it has been shown that acute ZIKV infection is associated with increased IFN-α levels in the blood sera of pregnant women [45]. Human infections with other related flaviviruses (dengue virus and West Nile virus) were also associated with increased IFN-α levels in the blood plasma/sera [86,87]. The relevance of IFN-α sequelae identified in porcine offspring affected with subclinical *in utero* ZIKV infection to sequelae in humans remains to be established. If ZIKV-induced IFN-α sequelae persist in human offspring, it might have dramatic health consequences. Excessive IFN-α levels were previously associated with severe viral infection [88], immune dysfunction [89], major depressive disorder [90,91], and dementia [92]. Importantly, cognitive impairment, severe neurological sequelae, seizures, prefrontal hypometabolism, and psychiatric syndromes were described in adult and pediatric patients treated with therapeutic doses of IFN-α [93–98]. Affected IFN-α responses during stress, specifically, the stress-induced IFN-α shutdown, might also influence the susceptibility of offspring to other infections. Interestingly, we recently demonstrated that similar to ZIKV infection [18,37], porcine circovirus 2 (a DNA virus of *Circoviridae* family which causes pathology and death in fetuses) *in utero* infection is associated with consistently increased fetal IFN-α levels (PCV2-positive fetuses—61.36±12.36 pg/ml of blood plasma; PCV2-negative—below the detection limit). This proof of concept study is in agreement with a hypothesis that type I IFNs is a common culprit of severe congenital viral infections and associated grave complications in fetuses [34]. In addition, we suggest that *in utero* inflammation induced by subclinical TORCH infections [99–108], including ZIKV, may evoke type I IFN pathology in offspring which can be targeted to reduce long-term sequelae.

In agreement with previous histological, magnetic resonance imaging, and computed tomography screening of porcine fetal brains [17,18], subclinical *in utero* ZIKV infection at mid and late gestational periods did not cause brain lesions in offspring. However, we found profound transcriptional changes in brains of offspring which did not show clinical signs of congenital Zika syndrome. We do not know whether two hours-long social stress which was induced two days before a necropsy could affect transcriptional signature in brains of Control and ZIKV offspring. Although, the considerable number of tested brain samples, the obvious difference of the whole genome expression between brains of Control and ZIKV offspring, and independent confirmation of the altered transcriptional profile in placental samples, clearly indicate effects of subclinical *in utero* infection.

Offspring brains were negative for ZIKV, suggesting that transcriptional changes in the PFC may be a persistent representation of earlier ZIKV replication in their brains during subclinical *in utero* infection. We believe that ZIKV caused brain infection in at least some offspring during their *in utero* fetal life and was subsequently cleared. In support, first, ZIKV-specific Abs in newborns detected at birth (P subgroup) (**Fig 2J**) postulated productive infection in developing fetuses during *in utero* life and most probably in fetal brains because fetal brain is the target for ZIKV in the porcine model [18,37]. Second, when porcine litters inoculated at the mid of gestation, ZIKV replicates in the fetal brain for at least 28 days [18,37] and cleared at 60 days [19]. The virus clearance from fetal brains of rhesus monkeys has been also suggested [109]. Third, affected type I IFN processes (**Fig 5**) and “defense response to virus” (FDR-adjusted *P* = 0.001) and “response to virus” (FDR-adjusted *P* = 0.009) (**S2B Table**) in brains of offspring, strongly suggest previous infection in the fetal brain. Finally, in offspring of the N subgroup, the lack of virus-specific Abs at birth (**Fig 2J**) does not rule out earlier subclinical fetal and fetal brain infection because ZIKV can persist in brains of porcine fetuses negative for Abs [18,37]. Even if ZIKV offspring in the N subgroup did not have virus replication in their internal organs during *in utero* life, most of them had infection in their individual amniotic membranes and placenta collected at birth (**Fig 2I; S1B and S1C Table**). Fetuses and fetal membranes form a conceptus, a fetal-fetal membrane unit, because fetal, amniotic membrane, and placental blood circulations are intimately interconnected. Replicating in individual amniotic membranes and placenta during persistent *in utero* infection, ZIKV may distantly affect the developing fetal brain. This was previously described in herpesvirus and malaria infections in mice and humans [110,111], where pathogens did not reach the fetus, but the inflammatory process in the placenta affected the normal fetal development. The role of immune responses to ZIKV at the maternal-fetal interface in birth defects has been also suggested [112]. In strong agreement, numerous biological processes, including vascular processes, were affected in the porcine placenta persistently infected with ZIKV (**Fig 9D**). Also, offspring of both N and P subgroups had the altered whole-genome expression signature in brains (**Fig 7**). In the fetal pig model of ZIKV infection, the cumulative negative effects originated in the developing fetal brain, fetal membranes, and placenta are also possible. Alternative animal model approaches are required to discriminate the effects of local ZIKV replication in the fetal brain and placenta on health in offspring.

Using RNA-seq, we defined sex-specific transcriptional changes in brains of affected porcine offspring, and subsequently confirmed sex-specific responses in infected placental samples. It is well-recognized that male fetuses are more vulnerable in affected pregnancies, with more adverse long-term outcomes occurring after birth [66,113]. Accordingly, ZIKV-affected male piglets demonstrated more dramatic molecular pathology both in the brain and placenta. In the brains, most affected biological processes related to neuropathology were represented by male offspring. In the placenta, a higher number of differentially expressed genes was showed by male offspring (**Fig 9B** and **9C**). Our findings are in agreement with a study in mice, where male offspring affected with mild ZIKV infection showed higher risks of developing neurocognitive disorders [12]. Moreover, in a recent study on the prospective human cohort of ZIKV-exposed children, male gender was identified as a potential predictor of delayed developmental alterations [7]. Observed behavioral differences between groups during social stress also had a sex-specific trend (**Fig 10**). Zika virus-affected male offspring showed more aggressive social behavior than affected female offspring as represented by a higher percentage of initiated and won fights (**Fig 10**). Altered behavior conveyed by ZIKV-affected porcine offspring during stress is in high agreement with findings in macaques [14], where postnatal ZIKV infection of infants resulted in altered functional connectivity between brain areas involved in emotional behavior and arousal functions, as well as in distinct alterations in the species-typical emotional reactivity to acute stress. It is difficult to compare our sex-specific data in pigs with the macaque model study due to different experimental approaches [14]. While ZIKV-affected offspring showed the trend to behavioral differences (**Fig 10**), the statistical significance was not attained in both female and male animals, which is not surprising in the context of subclinical *in utero* infection and silent pathology. Delayed neurodevelopmental abnormalities identified at the only second year of life have been described in the most recent study on the prospective human cohort of ZIKV-exposed children [7]. Thus, to further confirm and better understand behavioral sequelae in the porcine model, its functional connection to molecular pathology in the brain and placenta, and the relevance of the model to study delayed childhood neurodevelopment described in affected human offspring [7], future experiments should utilize the larger sample size, the longer observational periods, and a broader panel of standardized methods for behavioral and cognitive research in pigs [114–119].

Collectively, our results provide strong evidence for long-term silent immunopathology and sex-specific brain molecular pathology in porcine offspring and novel insights into pathogenesis of subclinical *in utero* ZIKV infection. We also demonstrated that subclinical prenatal ZIKV exposure and postnatal social stress may cumulatively induce immunopathology in affected offspring. These findings should encourage further efforts to better understand silent pathology in fetuses and offspring, monitor ZIKV-affected human cohorts, and develop strategies to prevent and alleviate long-term sequelae.

## Materials and methods

### Virus

We used low-passage, contemporary, Asian-lineage Zika virus (ZIKV) strain PRVABC59 [GenBank: KU501215.1] isolated from human serum specimen (Puerto Rico, 2015) [120]. After two passages on C6/36 cells, cell culture media containing ZIKV was centrifuged (12,000g, 20 min, +4°C), and the supernatant was collected. Media from virus-negative C6/36 cells was used for mock-inoculation. The absence of mycoplasma contamination in all inoculums and cell cultures was confirmed using LookOut Mycoplasma PCR Detection Kit (Sigma-Aldrich).

### Animal experimental design

Animal experiments were performed in strict accordance with the Canadian Council on Animal Care guidelines for humane animal use. All animal protocols were approved by the University of Saskatchewan's Animal Research Ethics Board. Six, pregnancy-matched Landrace-cross pigs, were obtained from a high-health status herd free for porcine reproductive and respiratory syndrome virus and porcine parvovirus (viruses which can cause fetal infection in pigs). To exclude porcine circovirus 2 (PCV2) *in utero* infection (another pig virus which can cause infection in fetuses) we tested blood plasma collected from all newborns at birth, before feeding colostrum, for PCV2 antibodies (Ab) [121]. All samples were negative for anti-PCV2 Ab. Pregnant pigs were housed at the Vaccine and Infectious Disease Organization-International Vaccine Centre (VIDO-InterVac) level 3 facilities. Pregnant pigs were randomly assigned into control (three animals) and ZIKV (three animals) groups and housed in identical, but isolated rooms. Housing conditions and diet were the same for all sows and offspring.

*In utero* inoculation was performed at 53–54 gestation days (gd) (the total duration of porcine pregnancy is 114–115 days) as previously described [18] with some modifications. Briefly, to establish subclinical infection, we manipulated only two conceptuses per pregnant pig. Two conceptuses from three experimental pigs (pig #109, #335, #409) were inoculated intraperitoneally + intra-amniotic (IP+IA; 100 μl+100 μl) with 10^5^ TCID_50_ (tissue culture infectious dose with 50% endpoint) of ZIKV. Two conceptuses from three control pigs (pigs #122, #179, #720) were inoculated with virus-free media. For precise inoculation, we used an ultrasound-guided technique which verifies fetal viability before and after injection by visualizing heart beating (**S1 Video**). Two fetuses close to the uterine bifurcation (**S1 Fig**) were directly inoculated. This fetal location was selected in order to maximize the virus spread within the uterus. Fetuses in the only one uterine horn were inoculated to reduce manipulation.

We collected blood samples (in sterile EDTA tubes with vacutainer blood-sampling system (BD)) from mothers at 53–54 and 112 gestation days (at 0 and 59 days post-fetal inoculation), on the day of parturition, and 14 and 21 days after parturition. After blood centrifugation (2,000g, 20 min, +4°C) plasma was aliquoted and immediately frozen (-80°C). Colostrum was collected at delivery day, centrifuged (2,000g, 20 min, +4°C), and immediately frozen at -80°C.

Births were monitored closely. Rejected after birth placental tissues and individual amniotic membranes surrounding each newborn were collected and immediately frozen on dry ice or preserved in 10% buffered formalin. At birth (day 0), we tagged piglets, recorded gender, and measured cranium, body dimensions and body weight. After birth, piglets were monitored for 36 days for clinical signs by veterinarians. Placental, amniotic membrane and brain tissues from stillborn and weak piglets were tested for ZIKV. Weak piglets were euthanized at day 2 after birth and excluded from the study (**S1A Table**). Mothers were separated from piglets at 21 days after birth. Piglets were weighed at birth and at 32 days after birth. Blood from piglets was collected at 0 (immediately after birth, before the first colostrum uptake), 14, 21, 32, and 35 days after birth. Blood was collected in sterile EDTA tubes with vacutainer blood-sampling system (BD) by puncture of the vena cava. After blood centrifugation (2,000g, 20 min, +4°C) plasma was aliquoted and immediately frozen (-80°C).

Piglets were euthanized at 37 days of age. Animals were euthanized by licensed veterinarians with an anesthetic overdose followed by exsanguination: After injecting the anesthetic, complete unconsciousness was confirmed by loss of pedal and palpebral reflexes and pigs were rapidly exsanguinated to ensure a quick death. This method minimizes animal distress, and is consistent with the recommendations of the Panel on Euthanasia of the American Veterinary Medical Association and approved by the University of Saskatchewan's Animal Research Ethics Board. At euthanasia, we weighted piglets. Brains were removed, weighed, and preserved in liquid nitrogen (left hemisphere) or formalin (right hemisphere) within 4–5 minutes after euthanasia. To avoid the effects of circadian rhythms on gene expression in the brain, animals from both groups were sampled at the same time within a short period.

### Individual activity in offspring

Cameras were mounted above the home pens with mothers and offspring for continuous video recording. Behavior of each piglet was recorded to determine whether *in utero* ZIKV infection affects individual activity in a regular environment, i.e., in a home pen in the presence of their mother (at 20 days after birth, the whole day), during a novel situation in a familiar environment, i.e., after removal of the mother from the home pen (the second half (12:00 pm -12:00 am) of the 21^st^ day), and at a later time point (at 22 days, the whole day) as previously described [79]. We analyzed video recordings and quantified the activity of the whole litter using sampling at 5-min intervals. The number of lying down, and sitting events (passive behavior), and the number of standing, walking, playing, interacting, and running events (active behavior) was registered for each litter [79]. The number of active piglets was divided by litter size and expressed as a percentage at each moment of observations [79].

### The mixing test

The mixing test [76–79] was performed at 35 days after birth to determine effects of subclinical *in utero* ZIKV infection on blood cytokine levels and aggressive behavior in a stressful environment—during a social confrontation with an unfamiliar piglet. We used following criteria to form mixing pairs: (**i**) A piglet from the ZIKV group was mixed with a piglet from the control group; (**ii**) males were mixed with males, females with females; (**iii**) the piglets' body weight differences did not exceed 1 kg.

Mixing pairs were individually marked with numbers on their backs, relocated to the mixing room and simultaneously released into individual unfamiliar mixing pens (2.5 m x 0.7 m). Cameras were mounted above each pen. The room was left, and videos were recorded for 120 min, as most fights between unfamiliar pigs occur during the first few hours after mixing [58,122,123]. Then immediately after the mixing test, we collected blood samples as described above. We used video recordings to score fighting activities (**S2 Video**). A fight, i.e., a ‘bout’ of fighting, was defined as a period of time lasting at least 10 s during which (**i**) two pigs showed close physical contact and (**ii**) five or more head knocks or bites were shown by one or both pigs. A fight was deemed to have ended when aggressive acts ceased after the retreat of one or both pigs and pigs were staying separated for 60 s or more. For every fight, we scored the initiator, i.e., the pig that first bites or head knocks. In addition, we recorded the winner and loser of every fight. The pig that first stopped fighting, retreated, turned away from its opponent or tried to flee was considered to be the loser of the fight. From these observations, we calculated the ratio of initiated fights and the ratio of winners within the group. Three blinded investigators performed analyses of behavioral data (**S1G Table**). Observations were compared (initiators: Fleiss’ kappa = 0.81, winners: Fleiss’ kappa = 0.98) and averaged both in males and females [124].

### Sample testing

RNA extraction, RT-qPCR assays, serology, Bio-Plex assay, cortisol assay, histology, and ZIKV-specific *in situ* hybridization were performed as previously described [18,19,75,125–131]. The sandwich ELISA (LSBio LS-F23292) to quantify porcine IFN-*β* in blood plasma samples was performed as per the manufacturer's instructions. All details on sample testing are provided in **S1 Appendix**.

### RNA-seq and bioinformatics

RNA was isolated using TRIzol (Thermo Fisher Scientific) lysis and extraction, and then cleaned using Total RNA Purification Kit (Norgen Biotek). RNA was assessed on a Bioanalyzer and all samples had RNA Integrity above 8.5. Complementary DNA (cDNA) libraries for sequencing were prepared using NEBNext Ultra II Directional RNA Library Prep Kit for Illumina with rRNA depletion (New England Biolabs). Libraries were sequenced on a NovaSeq as paired-end reads using the 150 base read kit. Over the whole experiment, there was an average of 26 million paired-end reads per sample. Sequencing data were mapped and quantified using the pseudo alignment method of Kallisto [132]. A sequence database of coding and non-coding transcripts was generated from ENSEMBL *Sus scrofa* 11.1. The count table was assembled using tximport in R and normalized using EdgeR then converted into a normal distribution using the voom function and differential expression was calculated using linear and Bayes models as part of the R package limma. Gene set enrichment was done using human annotation of Gene Ontology using the R function camera.

Confirmation of sex in placental samples was accomplished using the expression of the Y chromosome and expression of the X chromosome gene KDM6A as it escapes X inactivation and gives a higher signal in females. Correction for sex was done directly in the linear model by setting sex as a variable in the model. Before visualization corrected expression values were generated using the R function remove BatchEffects for data represented in heatmaps (generated using the R package pheatmap) or principal component plots (generated using the R function princomp and the ggplot2 package).

The set enrichment results from camera were graphed in Cytoscape using the EnrichmentMap plugin. All networks were generated using a Jaccard + Overlap with a cutoff of 0.375 and a Combined Constant of 0.5. Sub-networks were discovered using GLay cluster and annotated using the WordCloud plugin of the top 4 words with a bonus of 8 for word co-occurrence. Gene expression data are provided in **S2 Table**. An accession number for RNA-seq data is PRJNA573521 in NCBI BioProject.

### Statistical analysis

We used GraphPad PRISM7 software (GraphPad Software Inc., San Diego, CA, USA) for statistical analyses. Results were considered significantly different when *P* < 0.05. All data were expressed as mean ± standard deviation (M ± SD). All raw animal data for this study are provided in **S1 Table**. Prevalence of stillborn and weak piglets was compared using Pearson’s chi-squared test with Yates’ correction for continuity. Body and brain weights and body dimensions were compared with Mann-Whitney *U*-test. Maternal cytokines levels between control and ZIKV groups and within groups were compared with Bonferroni-corrected repeated measures analysis of variance (rANOVA). IFN-α levels in offspring blood plasma sampled at birth, 14, 21, and 32 days after birth were compared between control and ZIKV groups with Mann-Whitney *U*-test.

Unpaired Student’s t-test was used to analyze cortisol in hair samples. IFN-α levels in blood plasma sampled before and after the mixing test were compared between control and ZIKV groups by ANOVA with Tukey’s post-test. We also assessed fold change between IFN-α levels before and after the mixing test within N and P subgroups and in sex subgroups—Mann-Whitney U-test. In the mixing test, within each pair, we quantified a percentage of fights initiated and a percentage of fights won by control piglet or ZIKV piglet. The percentage of initiated and won fights—individually for males and females—were compared against the expected value (50% versus 50%) in control versus ZIKV groups with two-sided Wilcoxon signed-rank test.

## Supporting information

S1 Appendix Supplementary materials and methods. (DOCX)

S1 Fig A porcine uterus and *in utero* inoculation of fetuses with ZIKV. (**A**) A porcine uterus consists of the uterine body and two horns. (**B**) Each horn contains multiple fetuses with each fetus possessing individual amniotic membrane and placenta. Two fetuses (highlighted in red) in each pregnant pig were directly inoculated with ZIKV or control media. (**C**) Afterward, ZIKV spreads between siblings within the horn containing inoculated fetuses and between fetuses in the opposite horn. (**D**) As a result, most conceptuses (a fetus with fetal membranes) within the uterus are infected. *In utero* ZIKV infection kinetics and *in utero* ZIKV tropism in the porcine model is comprehensively described in previous publications [17–19,37].
(TIF)

S2 Fig Cytokine levels in maternal blood plasma. Circles represent data from individual sows. Boxes represent the highest and lowest observations. A horizontal line inside the box is the mean. An asterisk (*) represents a statistically significant difference (*P* < 0.05) between control and ZIKV groups. An arrowhead (▲) represents statistically significant difference within groups, versus day 0. Dpi–days post-inoculation, gd–gestation days. The dotted line represents LOQ. See raw data in **S1D Table** for individual values.
(TIF)

S3 Fig Transcriptional changes in the prefrontal cortex of offspring affected with subclinical *in utero* ZIKV infection (all Control offspring versus all ZIKV offspring). Heatmaps of 310 upregulated (**A**) and 143 downregulated (**B**) genes with FDR-adjusted *P* < 0.05. X and Y axes represent sample identification and genes, respectively. #122, #179, and #720—control litters; #109, #335, #409 –ZIKV litters. See raw data in **S2A Table** for individual gene values. ViralPCR—represents viral loads in amniotic membranes (**S1B Table**) (**C**) Enrichment plots of gene sets of “response to type I interferon” (FDR-adjusted *P* = 0.0026), “positive regulation of type I interferon production” (FDR-adjusted *P* = 0.026), “regulation of type I interferon production” (FDR-adjusted *P* = 0.011) and “response to interferon beta” (FDR-adjusted *P* = 0.08) GO processes (**S2B Table**). (**D**) Enrichment plot of gene sets of “response to corticosteroid” GO process (FDR-adjusted *P* = 0.03) (**S2B Table**). (**E**) Chronic cortisol in offspring hair collected at necropsy. Whiskers denote 95% confidence interval. See raw data in **S1F Table** for individual values.
(TIF)

S4 Fig Kinetics of IFN-α in the blood of ZIKV-affected porcine fetuses and offspring. IFN-α levels (M±SE) were measured in the blood plasma of ZIKV-affected and control porcine fetuses and offspring. Data for the fetal period (at 78 gestation days, gd) were compiled from our published study [18], where 53 virus-infected and 22 control fetuses were tested. Data for 110 gd are from study where 14 virus-infected and 16 control fetuses were tested (**S1C Table**). Elevated IFN-α levels at 78 gd were significantly higher in ZIKV infected fetuses (*P* = 0.0068, Mann-Whitney test) [18].
(TIF)

S1 Table Birth outcomes, virology, immunology results and the mixing test. (XLSX)

S2 Table RNA-seq data. (XLSB)

S1 Video Ultrasound-guided fetal inoculation. To confirm fetal viability, fetal heart beating was verified. First, a needle was inserted into the fetal peritoneal cavity for intraperitoneal (IP) injection. Second, the needle was pulled into the amniotic cavity for intraamniotic (IA) injection. After injections, fetal viability was confirmed by heart beating. The left panel represents the original video. The right panel is the same video with descriptive information. The video footage is slowed down (1.5x) for better perception.
(MP4)

S2 Video Aggressive behavior during a social confrontation with an unfamiliar piglet. Video footage of the mixing test demonstrates the social confrontation between Control and ZIKV piglets, the fight initiator (piglet #12), typical fight (piglets #8 and #10) and the winner (piglet #8).
(MP4)

## References

[1] Wheeler AC, Ventura CV, Ridenour T, Toth D, Nobrega LL, Silva de Souza Dantas LC, et al Skills attained by infants with congenital Zika syndrome: Pilot data from Brazil. Gopichandran V, editor. PLoS One. 2018;13: e0201495 10.1371/journal.pone.0201495 30048541 PMC6062124

[2] Brasil P, Pereira JP, Moreira ME, Ribeiro Nogueira RM, Damasceno L, Wakimoto M, et al Zika Virus Infection in Pregnant Women in Rio de Janeiro. N Engl J Med. 2016;375: 2321–2334. 10.1056/NEJMoa1602412 26943629 PMC5323261

[3] de Araújo TVB, Rodrigues LC, de Alencar Ximenes RA, de Barros Miranda-Filho D, Montarroyos UR, de Melo APL, et al Association between Zika virus infection and microcephaly in Brazil, January to May, 2016: preliminary report of a case-control study. The Lancet Infectious Diseases. 2016 10.1016/S1473-3099(16)30318-8 PMC7617035 27641777

[4] Silva AAM, Ganz JSS, Sousa PS, Doriqui MJR, Ribeiro MRC, Branco MRFC, et al Early growth and neurologic outcomes of infants with probable congenital Zika virus syndrome. Emerg Infect Dis. 2016;22: 1953–1956. 10.3201/eid2211.160956 27767931 PMC5088045

[5] Nogueira ML, Nery Júnior NRR, Estofolete CF, Bernardes Terzian AC, Guimarães GF, Zini N, et al Adverse birth outcomes associated with Zika virus exposure during pregnancy in São José do Rio Preto, Brazil. Clin Microbiol Infect. 2018;24: 646–652. 10.1016/j.cmi.2017.11.004 29133154

[6] Vianna RAO, Lovero KL, Oliveira SA, Fernandes AR, Santos TCS, Lima LCSS, et al Children Born to Mothers with Rash During Zika Virus Epidemic in Brazil: First 18 Months of Life. J Trop Pediatr. 2019; 10.1093/tropej/fmz019 31006031 PMC7962762

[7] Nielsen-Saines K, Brasil P, Kerin T, Vasconcelos Z, Gabaglia CR, Damasceno L, et al Delayed childhood neurodevelopment and neurosensory alterations in the second year of life in a prospective cohort of ZIKV-exposed children. Nat Med. 2019; 10.1038/s41591-019-0496-1 31285631 PMC6689256

[8] Adams Waldorf KM, Olson EM, Nelson BR, Little MTE, Rajagopal L. The Aftermath of Zika: Need for Long-Term Monitoring of Exposed Children. Trends Microbiol. 2018; 10.1016/j.tim.2018.05.011 29960747 PMC6136144

[9] Subissi L, Dub T, Besnard M, Mariteragi-Helle T, Nhan T, Lutringer-Magnin D, et al Zika virus infection during pregnancy and effects on early childhood development, French Polynesia, 2013–2016. Emerg Infect Dis. 2018;24: 1850–1858. 10.3201/eid2410.172079 30226164 PMC6154169

[10] Adams Waldorf KM, Nelson BR, Stencel-Baerenwald JE, Studholme C, Kapur RP, Armistead B, et al Congenital Zika virus infection as a silent pathology with loss of neurogenic output in the fetal brain. Nat Med. 2018;24: 368–374. 10.1038/nm.4485 29400709 PMC5839998

[11] Adams Waldorf KM, Stencel-Baerenwald JE, Kapur RP, Studholme C, Boldenow E, Vornhagen J, et al Fetal brain lesions after subcutaneous inoculation of Zika virus in a pregnant nonhuman primate. Nat Med. 2016;22: 1256–1259. 10.1038/nm.4193 27618651 PMC5365281

[12] Stanelle-Bertram S, Walendy-Gnirß K, Speiseder T, Thiele S, Asante IA, Dreier C, et al Male offspring born to mildly ZIKV-infected mice are at risk of developing neurocognitive disorders in adulthood. Nat Microbiol. 2018; 10.1038/s41564-018-0236-1 30202017

[13] Paul AM, Acharya D, Neupane B, Thompson EA, Gonzalez-Fernandez G, Copeland KM, et al Congenital Zika Virus Infection in Immunocompetent Mice Causes Postnatal Growth Impediment and Neurobehavioral Deficits. Front Microbiol. 2018;9: 2028 10.3389/fmicb.2018.02028 30210488 PMC6124374

[14] Mavigner M, Raper J, Kovacs-Balint Z, Gumber S, O’Neal JT, Bhaumik SK, et al Postnatal Zika virus infection is associated with persistent abnormalities in brain structure, function, and behavior in infant macaques. Sci Transl Med. 2018;10: eaao6975 10.1126/scitranslmed.aao6975 29618564 PMC6186170

[15] Giovanoli S, Engler H, Engler A, Richetto J, Voget M, Willi R, et al Stress in Puberty Unmasks Latent Neuropathological Consequences of Prenatal Immune Activation in Mice. Science. 2013;339: 1095–1099. 10.1126/science.1228261 23449593

[16] Straley ME, Van Oeffelen W, Theze S, Sullivan AM, O’Mahony SM, Cryan JF, et al Distinct alterations in motor & reward seeking behavior are dependent on the gestational age of exposure to LPS-induced maternal immune activation. Brain Behav Immun. 2017;63: 21–34. 10.1016/j.bbi.2016.06.002 27266391

[17] Wichgers Schreur PJ, Van Keulen L, Anjema D, Kant J, Kortekaas J. Microencephaly in fetal piglets following in utero inoculation of Zika virus. Emerg Microbes Infect. 2018;7: 42 10.1038/s41426-018-0044-y 29593256 PMC5874248

[18] Darbellay J, Cox B, Lai K, Delgado-Ortega M, Wheler C, Wilson D, et al Zika Virus Causes Persistent Infection in Porcine Conceptuses and may Impair Health in Offspring. EBioMedicine. 2017;25: 73–86. 10.1016/j.ebiom.2017.09.021 29097124 PMC5704061

[19] Trus I, Darbellay J, Huang Y, Gilmour M, Safronetz D, Gerdts V, et al Persistent Zika virus infection in porcine conceptuses is associated with elevated in utero cortisol levels. Virulence. 2018;9: 1338–1343. 10.1080/21505594.2018.1504558 30058440 PMC7000198

[20] Ibrahim Z, Busch J, Awwad M, Wagner R, Wells K, Cooper DKC. Selected physiologic compatibilities and incompatibilities between human and porcine organ systems. Xenotransplantation. 2006;13: 488–499. 10.1111/j.1399-3089.2006.00346.x 17059572

[21] Goco RV, Kress MB, Brantigan OC. Comparison of Mucus Glands in the Tracheobronchial Tree of Man and Animals. Ann N Y Acad Sci. 1963;106: 555–571. 10.1111/j.1749-6632.1963.tb16665.x 13963227

[22] Pabst R, Binns RM. The immune system of the respiratory tract in pigs. Vet Immunol Immunopathol. 1994;43: 151–156. 10.1016/0165-2427(94)90131-7 7856047

[23] Dawson HD, Loveland JE, Pascal G, Gilbert JGR, Uenishi H, Mann KM, et al Structural and functional annotation of the porcine immunome. BMC Genomics. 2013;14: 332 10.1186/1471-2164-14-332 23676093 PMC3658956

[24] Dawson HD, Smith AD, Chen C, Urban JF. An in-depth comparison of the porcine, murine and human inflammasomes; lessons from the porcine genome and transcriptome. Vet Microbiol. 2017;202: 2–15. 10.1016/j.vetmic.2016.05.013 27321134

[25] Bendixen E, Danielsen M, Larsen K, Bendixen C. Advances in porcine genomics and proteomics-a toolbox for developing the pig as a model organism for molecular biomedical research. Briefings Funct Genomics Proteomics. 2010;9: 208–219. 10.1093/bfgp/elq004 20495211

[26] Lunney JK. Advances in swine biomedical model genomics. Int J Biol Sci. 2007;3: 179–184. 10.7150/ijbs.3.179 17384736 PMC1802015

[27] Meurens F, Summerfield A, Nauwynck H, Saif L, Gerdts V. The pig: A model for human infectious diseases. Trends Microbiol. 2012;20: 50–57. 10.1016/j.tim.2011.11.002 22153753 PMC7173122

[28] Dickerson JW, Dobbing J. Prenatal and postnatal growth and development of the central nervous system of the pig. Proc R Soc London Ser B, Biol Sci. 1967;166: 384–395. 10.1098/rspb.1967.0002 24796035

[29] Rothkötter HJ, Sowa E, Pabst R. The pig as a model of developmental immunology. Hum Exp Toxicol. 2002;21: 533–536. 10.1191/0960327102ht293oa 12458912

[30] Dobbing J, Sands J. Comparative aspects of the brain growth spurt. Early Hum Dev. 1979;3: 79–83. 10.1016/0378-3782(79)90022-7 118862

[31] Pond WG, Boleman SL, Fiorotto ML, Ho H, Knabe DA, Mersmann HJ, et al Perinatal ontogeny of brain growth in the domestic pig. Proc Soc Exp Biol Med. 2000;223: 102–108. 10.1046/j.1525-1373.2000.22314.x 10632968

[32] Honein MA, Dawson AL, Petersen EE, Jones AM, Lee EH, Yazdy MM, et al Birth defects among fetuses and infants of US women with evidence of possible Zika virus infection during pregnancy. JAMA—J Am Med Assoc. 2017;317: 59–68. 10.1001/jama.2016.19006 27960197

[33] Jagger BW, Miner JJ, Cao B, Arora N, Smith AM, Kovacs A, et al Gestational Stage and IFN-λ Signaling Regulate ZIKV Infection In Utero. Cell Host Microbe. 2017;22: 366–376.e3. 10.1016/j.chom.2017.08.012 28910635 PMC5647680

[34] Yockey LJ, Jurado KA, Arora N, Millet A, Rakib T, Milano KM, et al Type I interferons instigate fetal demise after Zika virus infection. Sci Immunol. 2018;3: eaao1680 10.1126/sciimmunol.aao1680 29305462 PMC6049088

[35] Vermillion MS, Lei J, Shabi Y, Baxter VK, Crilly NP, McLane M, et al Intrauterine Zika virus infection of pregnant immunocompetent mice models transplacental transmission and adverse perinatal outcomes. Nat Commun. 2017;8: 14575 10.1038/ncomms14575 28220786 PMC5321801

[36] Antonson AM, Radlowski EC, Lawson MA, Rytych JL, Johnson RW. Maternal viral infection during pregnancy elicits anti-social behavior in neonatal piglet offspring independent of postnatal microglial cell activation. Brain Behav Immun. 2017;59: 300–312. 10.1016/j.bbi.2016.09.019 27650113

[37] Udenze D, Trus I, Berube N, Gerdts V, Karniychuk U. The African strain of Zika virus causes more severe in utero infection than Asian strain in a porcine fetal transmission model. Emerg Microbes Infect. 2019;8: 1098–1107. 10.1080/22221751.2019.1644967 31340725 PMC6711198

[38] Tayade C, Black GP, Fang Y, Croy BA. Differential Gene Expression in Endometrium, Endometrial Lymphocytes, and Trophoblasts during Successful and Abortive Embryo Implantation. J Immunol. 2006;176: 148–156. 10.4049/jimmunol.176.1.148 16365405

[39] Vanderhaeghe C, Dewulf J, De Vliegher S, Papadopoulos GA, de Kruif A, Maes D. Longitudinal field study to assess sow level risk factors associated with stillborn piglets. Anim Reprod Sci. 2010;120: 78–83. 10.1016/j.anireprosci.2010.02.010 20346603

[40] Šterzl J, Rejnek J, Trávníček J. Impermeability of pig placenta for antibodies. Folia Microbiol (Praha). 1966;11: 7–10. 10.1007/BF02877148 4957967

[41] Waysbort A, Giroux M, Mansat V, Teixeira M, Dumas JC, Puel J. Experimental study of transplacental passage of alpha interferon by two assay techniques. Antimicrob Agents Chemother. 1993;37: 1232–1237. 10.1128/aac.37.6.1232 8328774 PMC187945

[42] Šplíchal I, Řeháková Z, Šinkora M, Šinkora J, Trebichavský I, Laude H, et al In vivo study of interferon-alpha-secreting cells in pig foetal lymphohaematopoietic organs following in utero TGEV coronavirus injection. Res Immunol. 1997;148: 247–256. 10.1016/s0923-2494(97)80866-8 9300531 PMC7135581

[43] Li J, Mirnics K, Garbett K, Patterson PH, Smith SEP. Maternal Immune Activation Alters Fetal Brain Development through Interleukin-6. J Neurosci. 2007;27: 10695–10702. 10.1523/JNEUROSCI.2178-07.2007 17913903 PMC2387067

[44] Choi GB, Yim YS, Wong H, Kim S, Kim H, Kim S V., et al The maternal interleukin-17a pathway in mice promotes autism-like phenotypes in offspring. Science. 2016;351: 933–939. 10.1126/science.aad0314 26822608 PMC4782964

[45] Brasil P, Nielsen-Saines K, Jung JU, Chan Y, Chen W, Cheng G, et al Biomarkers and immunoprofiles associated with fetal abnormalities of ZIKV-positive pregnancies. JCI Insight. 2018;3 10.1172/jci.insight.124152 30385728 PMC6238739

[46] Russell E, Koren G, Rieder M, Van Uum S. Hair cortisol as a biological marker of chronic stress: Current status, future directions and unanswered questions. Psychoneuroendocrinology. 2012;37: 589–601. 10.1016/j.psyneuen.2011.09.009 21974976

[47] Davenport MD, Tiefenbacher S, Lutz CK, Novak MA, Meyer JS. Analysis of endogenous cortisol concentrations in the hair of rhesus macaques. Gen Comp Endocrinol. 2006;147: 255–61. 10.1016/j.ygcen.2006.01.005 16483573

[48] Karniychuk UU, Nauwynck HJ. Quantitative Changes of Sialoadhesin and CD163 Positive Macrophages in the Implantation Sites and Organs of Porcine Embryos/Fetuses During Gestation. Placenta. 2009;30: 497–500. 10.1016/j.placenta.2009.03.016 19410291

[49] Karniychuk UU, Saha D, Geldhof M, Vanhee M, Cornillie P, Van den Broeck W, et al Porcine reproductive and respiratory syndrome virus (PRRSV) causes apoptosis during its replication in fetal implantation sites. Microb Pathog. 2011;51: 194–202. 10.1016/j.micpath.2011.04.001 21511026

[50] Novakovic P, Harding JCS, Ladinig A, Al-Dissi AN, MacPhee DJ, Detmer SE. Relationships of CD163 and CD169 positive cell numbers in the endometrium and fetal placenta with type 2 PRRSV RNA concentration in fetal thymus. Vet Res. 2016;47: 76 10.1186/s13567-016-0364-7 27494990 PMC4974782

[51] Rosenberg AZ, Yu W, Hill DA, Reyes CA, Schwartz DA. Placental Pathology of Zika Virus: Viral Infection of the Placenta Induces Villous Stromal Macrophage (Hofbauer Cell) Proliferation and Hyperplasia. Arch Pathol Lab Med. 2017;141: 43–48. 10.5858/arpa.2016-0401-OA 27681334

[52] Caires-Júnior LC, Goulart E, Melo US, Araujo BSH, Alvizi L, Soares-Schanoski A, et al Discordant congenital Zika syndrome twins show differential in vitro viral susceptibility of neural progenitor cells. Nat Commun. 2018;9: 475 10.1038/s41467-017-02790-9 29396410 PMC5797251

[53] Linden V, Linden H Jr, Leal MC, Rolim Filho EL, Linden A, Aragão MFVV, et al Discordant clinical outcomes of congenital Zika virus infection in twin pregnancies. Arq Neuropsiquiatr. 2017;75: 381–386. 10.1590/0004-282X20170066 28658408

[54] Corry J, Arora N, Good CA, Sadovsky Y, Coyne CB. Organotypic models of type III interferon-mediated protection from Zika virus infections at the maternal–fetal interface. Proc Natl Acad Sci. 2017;114: 9433–9438. 10.1073/pnas.1707513114 28784796 PMC5584447

[55] Sun X, Hua S, Chen H-R, Ouyang Z, Einkauf K, Tse S, et al Transcriptional Changes during Naturally Acquired Zika Virus Infection Render Dendritic Cells Highly Conducive to Viral Replication. Cell Rep. 2017;21: 3471–3482. 10.1016/j.celrep.2017.11.087 29262327 PMC5751936

[56] Szaba FM, Tighe M, Kummer LW, Lanzer KG, Ward JM, Lanthier P, et al Zika virus infection in immunocompetent pregnant mice causes fetal damage and placental pathology in the absence of fetal infection. PLoS Pathog. 2018;14: e1006994 10.1371/journal.ppat.1006994 29634758 PMC5909921

[57] Hirsch AJ, Roberts VHJ, Grigsby PL, Haese N, Schabel MC, Wang X, et al Zika virus infection in pregnant rhesus macaques causes placental dysfunction and immunopathology. Nat Commun. 2018;9: 263 10.1038/s41467-017-02499-9 29343712 PMC5772047

[58] Meese GB, Ewbank R. The establishment and nature of the dominance hierarchy in the domesticated pig. Anim Behav. 1973;21: 326–334. 10.1016/S0003-3472(73)80074-0

[59] Shao Q, Herrlinger S, Zhu Y-N, Yang M, Goodfellow F, Stice SL, et al The African Zika virus MR-766 is more virulent and causes more severe brain damage than current Asian lineage and dengue virus. Development. 2017;144: 4114–4124. 10.1242/dev.156752 28993398 PMC5719247

[60] Coffey LL, Keesler RI, Pesavento PA, Woolard K, Singapuri A, Watanabe J, et al Intraamniotic Zika virus inoculation of pregnant rhesus macaques produces fetal neurologic disease. Nat Commun. 2018;9: 2414 10.1038/s41467-018-04777-6 29925843 PMC6010452

[61] Boulanger-Bertolus J, Pancaro C, Mashour GA. Increasing Role of Maternal Immune Activation in Neurodevelopmental Disorders. Front Behav Neurosci. 2018;12: 230 10.3389/fnbeh.2018.00230 30344483 PMC6182081

[62] Barker DJP. The fetal and infant origins of disease. Eur J Clin Invest. 1995;25: 457–463. 10.1111/j.1365-2362.1995.tb01730.x 7556362

[63] Blomström Å, Karlsson H, Gardner R, Jörgensen L, Magnusson C, Dalman C. Associations between Maternal Infection during Pregnancy, Childhood Infections, and the Risk of Subsequent Psychotic Disorder—A Swedish Cohort Study of Nearly 2 Million Individuals. Schizophr Bull. 2016;42: 125–133. 10.1093/schbul/sbv112 26303935 PMC4681563

[64] Spann MN, Monk C, Scheinost D, Peterson BS. Maternal Immune Activation During the Third Trimester Is Associated with Neonatal Functional Connectivity of the Salience Network and Fetal to Toddler Behavior. J Neurosci. 2018;38: 2877–2886. 10.1523/JNEUROSCI.2272-17.2018 29487127 PMC5852665

[65] da Paz VRF, Sequeira D, Pyrrho A. Infection by Schistosoma mansoni during pregnancy: Effects on offspring immunity. Life Sci. 2017;185: 46–52. 10.1016/j.lfs.2017.07.021 28754617

[66] Cheong JN, Wlodek ME, Moritz KM, Cuffe JSM. Programming of maternal and offspring disease: impact of growth restriction, fetal sex and transmission across generations. J Physiol. 2016;594: 4727–4740. 10.1113/JP271745 26970222 PMC5009791

[67] Garay PA, Hsiao EY, Patterson PH, McAllister AK. Maternal immune activation causes age- and region-specific changes in brain cytokines in offspring throughout development. Brain Behav Immun. 2013;31: 54–68. 10.1016/j.bbi.2012.07.008 22841693 PMC3529133

[68] Brenhouse HC, Schwarz JM. Immunoadolescence: Neuroimmune development and adolescent behavior. Neurosci Biobehav Rev. 2016;70: 288–299. 10.1016/j.neubiorev.2016.05.035 27260127 PMC5412135

[69] Györffy BA, Gulyássy P, Gellén B, Völgyi K, Madarasi D, Kis V, et al Widespread alterations in the synaptic proteome of the adolescent cerebral cortex following prenatal immune activation in rats. Brain Behav Immun. 2016;56: 289–309. 10.1016/j.bbi.2016.04.002 27058163

[70] Giovanoli S, Weber-Stadlbauer U, Schedlowski M, Meyer U, Engler H. Prenatal immune activation causes hippocampal synaptic deficits in the absence of overt microglia anomalies. Brain Behav Immun. 2016;55: 25–38. 10.1016/j.bbi.2015.09.015 26408796

[71] Meyer U, Feldon J, Fatemi SH. In-vivo rodent models for the experimental investigation of prenatal immune activation effects in neurodevelopmental brain disorders. Neurosci Biobehav Rev. 2009;33: 1061–1079. 10.1016/j.neubiorev.2009.05.001 19442688

[72] Lupien SJ, McEwen BS, Gunnar MR, Heim C. Effects of stress throughout the lifespan on the brain, behaviour and cognition. Nat Rev Neurosci. 2009;10: 434–445. 10.1038/nrn2639 19401723

[73] Le Pen G, Gourevitch R, Hazane F, Hoareau C, Jay TM, Krebs M-O. Peri-pubertal maturation after developmental disturbance: A model for psychosis onset in the rat. Neuroscience. 2006;143: 395–405. 10.1016/j.neuroscience.2006.08.004 16973297

[74] McCormick CM, Mathews IZ. Adolescent development, hypothalamic-pituitary-adrenal function, and programming of adult learning and memory. Prog Neuro-Psychopharmacology Biol Psychiatry. 2010;34: 756–765. 10.1016/j.pnpbp.2009.09.019 19782715

[75] Darbellay J, Lai K, Babiuk S, Berhane Y, Ambagala A, Wheler C, et al Neonatal pigs are susceptible to experimental Zika virus infection. Emerg Microbes Infect. 2017;6: e6 10.1038/emi.2016.133 28196970 PMC5322322

[76] Bolhuis JE, Schouten WGP, Schrama JW, Wiegant VM. Individual coping characteristics, aggressiveness and fighting strategies in pigs. Anim Behav. 2005;69: 1085–1091. 10.1016/j.anbehav.2004.09.013

[77] van der Staay FJ, De Groot J, Van Reenen CG, Hoving-Bolink AH, Schuurman T, Schmidt BH. Effects of Butafosfan on salivary cortisol and behavioral response to social stress in piglets. J Vet Pharmacol Ther. 2007;30: 410–416. 10.1111/j.1365-2885.2007.00884.x 17803732

[78] van der Staay FJ, de Groot J, Schuurman T, Korte SM. Repeated social defeat in female pigs does not induce neuroendocrine symptoms of depression, but behavioral adaptation. Physiol Behav. 2008;93: 453–460. 10.1016/j.physbeh.2007.10.002 17991496

[79] Kranendonk G, Hopster H, Fillerup M, Ekkel ED, Mulder EJH, Taverne MAM. Cortisol administration to pregnant sows affects novelty-induced locomotion, aggressive behaviour, and blunts gender differences in their offspring. Horm Behav. 2006;49: 663–672. 10.1016/j.yhbeh.2005.12.008 16488416

[80] Hansmann L, Groeger S, von Wulffen W, Bein G, Hackstein H. Human monocytes represent a competitive source of interferon-α in peripheral blood. Clin Immunol. 2008;127: 252–264. 10.1016/j.clim.2008.01.014 18342575

[81] Siegal FP, Kadowaki N, Shodell M, Fitzgerald-Bocarsly PA, Shah K, Ho S, et al The nature of the principal type 1 interferon-producing cells in human blood. Science. 1999;284: 1835–7. 10.1126/science.284.5421.1835 10364556

[82] Summerfield A, Guzylack-Piriou L, Schaub A, Carrasco CP, Tâche V, Charley B, et al Porcine peripheral blood dendritic cells and natural interferon-producing cells. Immunology. 2003;110: 440–9. 10.1111/j.1365-2567.2003.01755.x 14632641 PMC1783075

[83] Kasper C, Lübking A, Beelen DW, Dührsen U. Interferon alpha (IFN) treatment of bone marrow stroma inhibits haematopoesis. Leuk Res. 2004;28: 1217–1220. 10.1016/j.leukres.2004.03.012 15380348

[84] Essers MAG, Offner S, Blanco-Bose WE, Waibler Z, Kalinke U, Duchosal MA, et al IFNα activates dormant haematopoietic stem cells in vivo. Nature. 2009;458: 904–908. 10.1038/nature07815 19212321

[85] Prendergast ÁM, Kuck A, van Essen M, Haas S, Blaszkiewicz S, Essers MAG. IFNα-mediated remodeling of endothelial cells in the bone marrow niche. Haematologica. 2017;102: 445–453. 10.3324/haematol.2016.151209 27742772 PMC5394972

[86] Tobler LH, Cameron MJ, Lanteri MC, Prince HE, Danesh A, Persad D, et al Interferon and Interferon‐Induced Chemokine Expression Is Associated with Control of Acute Viremia in West Nile Virus–Infected Blood Donors. J Infect Dis. 2008;198: 979–983. 10.1086/591466 18729779 PMC7202400

[87] Kurane I, Innis BL, Nimmannitya S, Nisalak A, Ennis FA, Meager A. High Levels of Interferon Alpha in the Sera of Children with Dengue Virus Infection. Am J Trop Med Hyg. 1993;48: 222–229. 10.4269/ajtmh.1993.48.222 8447527

[88] Davidson S, Crotta S, McCabe TM, Wack A. Pathogenic potential of interferon αβ in acute influenza infection. Nat Commun. 2014;5: 3864 10.1038/ncomms4864 24844667 PMC4033792

[89] Cha L, Berry CM, Nolan D, Castley A, Fernandez S, French MA. Interferon-alpha, immune activation and immune dysfunction in treated HIV infection. Clin Transl Immunol. 2014;3: e10 10.1038/cti.2014.1 25505958 PMC4232062

[90] Mostafavi S, Battle A, Zhu X, Potash JB, Weissman MM, Shi J, et al Type I interferon signaling genes in recurrent major depression: Increased expression detected by whole-blood RNA sequencing. Mol Psychiatry. 2014;19: 1267–1274. 10.1038/mp.2013.161 24296977 PMC5404932

[91] Huckans M, Fuller B, Wheaton V, Jaehnert S, Ellis C, Kolessar M, et al A longitudinal study evaluating the effects of interferon-alpha therapy on cognitive and psychiatric function in adults with chronic hepatitis C. J Psychosom Res. 2015;78: 184–192. 10.1016/j.jpsychores.2014.07.020 25219976 PMC4435678

[92] Rho MB, Wesselingh S, Glass JD, McArthur JC, Choi S, Griffin J, et al A potential role for interferon-α in the pathogenesis of HIV-associated dementia. Brain Behav Immun. 1995;9: 366–377. 10.1006/brbi.1995.1034 8903853

[93] Shakil AO, Di Bisceglie AM, Hoofnagle JH. Seizures during alpha interferon therapy. J Hepatol. 1996;24: 48–51. 10.1016/s0168-8278(96)80185-1 8834024

[94] Lieb K, Engelbrecht MA, Gut O, Fiebich BL, Bauer J, Janssen G, et al Cognitive impairment in patients with chronic hepatitis treated with interferon alpha (IFNα): results from a prospective study. Eur Psychiatry. 2006;21: 204–210. 10.1016/j.eurpsy.2004.09.030 16632167

[95] Murray DM, Hensey OJ, O’Dwyer TP, King MD. Letter to the editor: Further evidence of neurological sequelae associated with interferon therapy in the pediatric population. Eur J Paediatr Neurol. 2000;4: 295–296. 10.1053/ejpn.2000.0388 11277372

[96] Schaefer M, Engelbrechta MA, Gut O, Fiebich BL, Bauer J, Schmidt F, et al Interferon alpha (IFNα) and psychiatric syndromes: A review. Prog Neuro-Psychopharmacology Biol Psychiatry. 2002;26: 731–746. 10.1016/S0278-5846(01)00324-4 12188106

[97] Juengling FD, Ebert D, Gut O, Engelbrecht MA, Rasenack J, Nitzsche EU, et al Prefrontal cortical hypometabolism during low-dose interferon alpha treatment. Psychopharmacology (Berl). 2000;152: 383–389. 10.1007/s002130000549 11140330

[98] Raison CL, Demetrashvili M, Capuron L, Miller AH. Neuropsychiatric adverse effects of interferon-alpha: recognition and management. CNS Drugs. 2005;19: 105–23. 10.2165/00023210-200519020-00002 15697325 PMC1255968

[99] Kieffer F, Thulliez P, Brézin A, Nobre R, Romand S, Yi-Gallimard E, et al [Treatment of subclinical congenital toxoplasmosis by sulfadiazine and pyrimethamine continuously during 1 year: apropos of 46 cases]. Arch Pediatr. 2002;9: 7–13. French. 10.1016/s0929-693x(01)00687-x 11865553

[100] Stagno S, Reynolds DW, Amos CS, Dahle AJ, McCollister FP, Mohindra I, et al Auditory and visual defects resulting from symptomatic and subclinical congenital cytomegaloviral and toxoplasma infections. Pediatrics. 1977;59: 669–78. 193086

[101] Diez B, Galdeano A, Nicolas R, Cisterna R. Relationship between the production of interferon-alpha/beta and interferon-gamma during acute toxoplasmosis. Parasitology. 1989;99 Pt 1: 11–5. 2508036 10.1017/s0031182000060972

[102] DeFilippis VR, Alvarado D, Sali T, Rothenburg S, Früh K. Human Cytomegalovirus Induces the Interferon Response via the DNA Sensor ZBP1. J Virol. 2010;84: 585–598. 10.1128/JVI.01748-09 19846511 PMC2798427

[103] Hoarau C, Ranivoharimina V, Chavet-Quéru MS, Rason I, Rasatemalala H, Rakotonirina G, et al [Congenital syphilis: update and perspectives]. Sante. 9: 38–45. French. 10210801

[104] Smoleniec JS, Pillai M, Caul EO, Usher J. Subclinical transplacental parvovirus B19 infection: an increased fetal risk? Lancet. 1994;343: 1100–1101. 10.1016/S0140-6736(94)90212-7 7909117

[105] Hayden GF, Herrmann KL, Buimovici-Klein E, Weiss KE, Nieburg PI, Mitchell JE. Subclinical congenital rubella infection associated with maternal rubella vaccination in early pregnancy. J Pediatr. 1980;96: 869–72. 10.1016/s0022-3476(80)80562-2 7365590

[106] Duran N, Yarkin F, Evruke C, Koksal F. Asymptomatic herpes simplex virus type 2 (HSV-2) infection among pregnant women in Turkey. Indian J Med Res. 2004;120: 106–10. 15347860

[107] Abbink P, Larocca RA, De La Barrera RA, Bricault CA, Moseley ET, Boyd M, et al Protective efficacy of multiple vaccine platforms against Zika virus challenge in rhesus monkeys. Science. 2016;353: 1129–1132. 10.1126/science.aah6157 27492477 PMC5237380

[108] Rasmussen SB, Sørensen LN, Malmgaard L, Ank N, Baines JD, Chen ZJ, et al Type I interferon production during herpes simplex virus infection is controlled by cell-type-specific viral recognition through Toll-like receptor 9, the mitochondrial antiviral signaling protein pathway, and novel recognition systems. J Virol. 2007;81: 13315–24. 10.1128/JVI.01167-07 17913820 PMC2168887

[109] Martinot AJ, Abbink P, Afacan O, Prohl AK, Bronson R, Hecht JL, et al Fetal Neuropathology in Zika Virus-Infected Pregnant Female Rhesus Monkeys. Cell. 2018;173: 1111–1122.e10. 10.1016/j.cell.2018.03.019 29606355 PMC5959775

[110] Cardenas I, Means RE, Aldo P, Koga K, Lang SM, Booth CJ, et al Viral infection of the placenta leads to fetal inflammation and sensitization to bacterial products predisposing to preterm labor. J Immunol. 2010;185: 1248–57. 10.4049/jimmunol.1000289 20554966 PMC3041595

[111] Desai M, ter Kuile FO, Nosten F, McGready R, Asamoa K, Brabin B, et al Epidemiology and burden of malaria in pregnancy. Lancet Infect Dis. 2007;7: 93–104. 10.1016/S1473-3099(07)70021-X 17251080

[112] Mor G. Placental Inflammatory Response to Zika Virus may Affect Fetal Brain Development. Am J Reprod Immunol. 2016;75: 421–422. 10.1111/aji.12505 26892436

[113] Eriksson JG, Kajantie E, Osmond C, Thornburg K, Barker DJP. Boys live dangerously in the womb. Am J Hum Biol. 2010;22: 330–335. 10.1002/ajhb.20995 19844898 PMC3923652

[114] Andersen AD, Sangild PT, Munch SL, van der Beek EM, Renes IB, Ginneken C van, et al Delayed growth, motor function and learning in preterm pigs during early postnatal life. Am J Physiol Integr Comp Physiol. 2016;310: R481–R492. 10.1152/ajpregu.00349.2015 26764054

[115] Gieling ET, Schuurman T, Nordquist RE, van der Staay FJ. The pig as a model animal for studying cognition and neurobehavioral disorders. Current Topics in Behavioral Neurosciences. 2011 pp. 359–383. 10.1007/7854_2010_112 21287323

[116] Kanitz E, Hameister T, Tuchscherer A, Tuchscherer M, Puppe B. Social Support Modulates Stress-Related Gene Expression in Various Brain Regions of Piglets. Front Behav Neurosci. 2016;10: 227 10.3389/fnbeh.2016.00227 27965550 PMC5126102

[117] Lind NM, Olsen AK, Moustgaard A, Jensen SB, Jakobsen S, Hansen AK, et al Mapping the amphetamine-evoked dopamine release in the brain of the Göttingen minipig. Brain Res Bull. 2005;65: 1–9. 10.1016/j.brainresbull.2004.08.007 15680539

[118] Lind NM, Arnfred SM, Hemmingsen RP, Hansen AK. Prepulse inhibition of the acoustic startle reflex in pigs and its disruption by D-amphetamine. Behav Brain Res. 2004;155: 217–222. 10.1016/j.bbr.2004.04.014 15364480

[119] van der Staay FJ, Pouzet B, Mahieu M, Nordquist RE, Schuurman T. The d-amphetamine-treated Göttingen miniature pig: An animal model for assessing behavioral effects of antipsychotics. Psychopharmacology (Berl). 2009;206: 715–729. 10.1007/s00213-009-1599-z 19626314 PMC2755106

[120] Lanciotti RS, Lambert AJ, Holodniy M, Saavedra S, del Carmen Castillo Signor L. Phylogeny of Zika virus in western Hemisphere, 2015. Emerg Infect Dis. 2016;22: 933–935. 10.3201/eid2205.160065 27088323 PMC4861537

[121] Saha D, Karniychuk UU, Huang L, Geldhof M, Vanhee M, Lefebvre DJ, et al Unusual outcome of in utero infection and subsequent postnatal super-infection with different PCV2b strains. Virol Sin. 2014;29 10.1007/s12250-014-3431-0 24950783 PMC8206427

[122] McGlone JJ. Influence of resources on pig aggression and dominance. Behav Processes. 1986;12: 135–144. 10.1016/0376-6357(86)90052-5 24897348

[123] Arey DS, Franklin MF. Effects of straw and unfamiliarity on fighting between newly mixed growing pigs. Appl Anim Behav Sci. 1995;45: 23–30. 10.1016/0168-1591(95)00600-W

[124] Fleiss JL. Measuring nominal scale agreement among many raters. Psychol Bull. 1971;76: 378–382. 10.1037/h0031619

[125] Xu MY, Liu SQ, Deng CL, Zhang QY, Zhang B. Detection of Zika virus by SYBR green one-step real-time RT-PCR. J Virol Methods. 2016;236: 93–97. 10.1016/j.jviromet.2016.07.014 27444120

[126] Faye O, Faye O, Dupressoir A, Weidmann M, Ndiaye M, Alpha Sall A. One-step RT-PCR for detection of Zika virus. J Clin Virol. 2008;43: 96–101. 10.1016/j.jcv.2008.05.005 18674965

[127] Nem de Oliveira Souza I, Frost PS, França J V., Nascimento-Viana JB, Neris RLS, Freitas L, et al Acute and chronic neurological consequences of early-life zika virus infection in mice. Sci Transl Med. 2018;10: eaar2749 10.1126/scitranslmed.aar2749 29875203

[128] Zupan M, Zanella AJ. Peripheral regulation of stress and fear responses in pigs from tail-biting pens. Rev Bras Zootec. 2017;46: 33–38. 10.1590/S1806-92902017000100006

[129] Turpin DL, Langendijk P, Chen TY, Lines D, Pluske JR. Intermittent suckling causes a transient increase in cortisol that does not appear to compromise selected measures of pigletwelfare and stress. Animals. 2016;6: 24 10.3390/ani6030024 26999224 PMC4810052

[130] Rault JL, Dunshea FR, Pluske JR. Effects of oxytocin administration on the response of piglets to weaning. Animals. 2015;5: 545–560. 10.3390/ani5030371 26479373 PMC4598693

[131] Macbeth BJ, Cattet MRL, Stenhouse GB, Gibeau ML, Janz DM. Hair cortisol concentration as a noninvasive measure of long-term stress in free-ranging grizzly bears (Ursus arctos): considerations with implications for other wildlife. Can J Zool. 2010;88: 935–949. 10.1139/z10-057

[132] Bray NL, Pimentel H, Melsted P, Pachter L. Near-optimal probabilistic RNA-seq quantification. Nat Biotechnol. 2016;34: 525–527. 10.1038/nbt.3519 27043002

