# Supplementary material for: Subclinical in utero Zika virus infection is associated with interferon alpha sequelae and sex-specific molecular brain pathology in asymptomatic porcine offspring
Source: PLoS Pathog. 2019 Nov 14;15(11):e1008038. doi: 10.1371/journal.ppat.1008038 (PMC6855438; doi:10.1371/journal.ppat.1008038)
Supplement: S1 Appendix — (DOCX) [file ppat.1008038.s001.docx]

**Supplementary** **Materials and Methods**

**RNA extraction and RT-PCR**

RNA from amniotic membranes, placenta, cerebrum (prefrontal cortex) and cerebellum was extracted using QIAamp Viral RNA Mini Kit (QIAGEN, USA) as previously described [1]. Before RNA extractions tissue samples were weighed and homogenized in 560 μl AVL lysis buffer (amniotic membranes, placenta) or 1 ml QIAzol Lysis Reagent (fetal cerebrum and cerebellum) using RNase-free stainless steel beads and TissueLyser II (QIAGEN, USA) operating for 5 min at 25 Hz.

All PCR reactions were conducted on the StepOne Plus platform (Life Technologies, USA) and analyzed using StepOne software version 2.3. ZIKV specific SYBR green-based one-step real-time reverse transcriptase quantitative polymerase chain reaction assay (RT-qPCR) was used for ZIKV RNA quantification [2]. One-Step RT-PCR (ref.: Bioline BIO-73005) reaction mixture (20 μl) consisted of 10 μl 2x SensiFAST SYBR Hi-ROX One-Step Mix, 0.4 μl RiboSafe RNase Inhibitor, 0.2 μl reverse transcriptase, 0.8 μl (400 nM) of each primer (ZIKV-F: 5′-AGGATCATAGGTGATGAAGAAAAGT-3′; ZIKV−R: 5′-CCTGACAACACTAAGATTGGTGC-3′), 3.8 μl nuclease-free water and 4 μl RNA. A reverse transcription step of 10 min at 45 °C and an enzyme activation step of 2 min at 95 °C were followed by 40 amplification cycles (5 s at 95 °C and 34 s at 60 °C). RNA from a stock of the ZIKV PRVABC59 strain ZIKV was used to generate a standard curve and quantify viral RNA loads. The results indicated that the standard curve had a wide dynamic range (10^2.0^-10^9.0^ copies/reaction) with the high linear correlation (R^2^ = 0.9997) between the cycle threshold (Ct) value and template concentration. The slope of the standard curve (-3.4351) corresponded to the 95.5% reaction efficiency level. Values from tissue samples were corrected for their weight and upon logarithmical transformation expressed as ZIKV RNA genome copies per g.

In addition to SYBR RT-qPCR, brain samples were tested in the probe-based one-step real-time RT-qPCR [3]. One-Step RT-qPCR (ref.: Bioline BIO-77005) reaction mixture (20 μl) consisted of 10 μl 2x SensiFAST Probe One-Step Mix, 0.4 μl RiboSafe RNase Inhibitor, 0.2 μl reverse transcriptase, 1.25 μl (625 nM) of each primer (ZF-9271-9297: 5′-AARTACACATACCARAACAAAGTGGT-3′, ZR-9352-9373: 5′-TCCRCTCCCYCTYTGGTCTTG-3′), 1 μl (500 nM) probe (ZP-NS5: 5′-FAM-CTYAGACCAGCTGAAR-BBQ-3′), 1.9 μl nuclease-free water and 4 μl RNA. A reverse transcription step of 10 min at 50 °C and an enzyme activation step of 2 min at 95 °C were followed by 60 amplification cycles (15 s at 95 °C and 60 s at 55 °C).

Persistent ZIKV replication in placental and amniotic membrane tissues was confirmed with ZIKV negative-strand RT-PCR [4]. cDNA was synthesized with SuperScript IV First-Strand Synthesis System (Invitrogen) using 10 pmole of ZIKV-835 forward primer (5’-TTGGTCATGATACTGCTGATTGC-3’) instead of random hexamer primers. Afterward, cDNA was amplified with primers (500 nM) (ZIKV-F1086: 5′-CCGCTGCCCAACACAAG-3′, ZIKV-R1162: 5′-CCACTAACGTTCTTTTGCAGACAT-3′), and probe (250 nM) (ZP-N: 5′-FAM-AGCCTACCT-ZEN-TGACAAGCAATCAGACACTCAA-IABkFQ-3′). An enzyme activation step of 5 min at 95 °C was followed by 60 amplification cycles (10 s at 95 °C and 20 s at 60 °C).

In all RT-PCR tests, we used Vero E6 cell culture media containing ZIKV as a positive RT-PCR control. As a negative control, we used samples from mock-inoculated and non-manipulated control fetuses. Strict precautions were taken to prevent PCR contamination. Aerosol-resistant filter pipette tips and disposable gloves were used. Kit reagent controls were included in every RNA isolation and RT-qPCR run.

**Serologic assay**

For quantification of ZIKV-specific IgG Ab immunoperoxidase monolayer assay (IPMA) was used as described before [1,5,6]. Briefly, Vero E6 cells in 96-well cell culture plates were inoculated with 50 μl media containing 2.3 log10 TCID_50_/ml ZIKV PRVABC59 strain and incubated (2h, +37 °C, 5% CO_2_). Then 100 μl of the culture medium was added and after incubation (72h, +37 °C, 5% CO_2_) plates were dried and stored at -20°C until use. For titrations plates were thawed and cells were fixed in 10% buffered formalin (30 min, RT). Cells were washed twice with 1x PBS (pH 7.2) and incubated with 100% methanol in the presence of 0.3% H_2_O_2_ (10 min, RT). Then plates were washed with PBS and two-fold [serial dilutions](https://www.sciencedirect.com/topics/medicine-and-dentistry/serial-dilution) of blood plasma or colostrum were added, followed by incubation (1h, +37 °C). Plates were washed three times with PBS containing 0.05% Tween 80 and 50 μl/well of rabbit anti-pig IgG conjugated with horseradish peroxidase (1:400; Abcam, USA) was added. After incubation (1h, +37 °C) and washing, color reaction was initiated by adding substrate solution (1 mM 3-amino-9-ethylcarbazole, 5% N,N-dimethylformamide, 50 mM Sodium Acetate (pH 5.0), 0.023% H_2_O_2_). The reaction was stopped by replacing the substrate with an acetate buffer, and ZIKV-specific staining was determined by examination with a microscope. The titers were defined as the reciprocal logarithm of 2 of the highest serum dilution. Blood plasma and colostrum from mock-inoculated and non-manipulated animals were used as negative controls.

**Bio-Plex assay**

Bio-Plex assay reagents are listed in the table below. We measured interleukin-1*β* (IL-1*β*), interleukin-6 (IL-6), interleukin-8 (IL-8), interleukin-10 (IL-10), interleukin-12 (IL-12), interleukin-13 (IL-13), interleukin-17A (IL-17A), transforming growth factor beta (TGF-*β*), tumor necrosis factor (TNF), interferon gamma (IFN-γ), and interferon alpha (IFN-α) in blood plasma as previously described [1,7]. Briefly, Bio-Plex bead coupling was performed as per the manufacturer's instructions. The multiplex assay was carried out in 96 well black plates (Grenier Bio-One Fluotrac 200). The beadsets conjugated with the capture antibodies were vortexed for 30 s followed by sonication for another 30 s to ensure total bead dispersal. The bead density was 1200 beads/μl in 1x PBS pH 7.4 supplemented with 1% bovine serum albumin (Sigma-Aldrich) and 0.05% sodium azide (Sigma-Aldrich)). 1 μl of each beadset was added to the diluent buffer (1x PBS pH 7.4 supplemented with 1% porcine serum and 0.05% sodium azide) for a total volume of 50 μl per well. The plate was then washed 2x using 100 μl washing buffer (1x PBS pH 7.4 with 0.05% Tween-20) and the BioRad Bio-Plex Pro II Wash Station. The protein standards with starting concentrations as indicated in the table below were mixed in the diluent buffer and 2.5 fold dilutions were made to produce the standard curve. 50 μl per well of each dilution was added to the plate. Samples were prediluted in diluent buffer (1:4) and 50 μl/well was added in duplicate. The plate was agitated at 800 rpm for 1 h at room temperature then washed (3x 150 μl washing buffer). Then 50 μl of the detection antibodies cocktail (consisting of the biotin-labeled antibodies diluted as indicated in the table below) was added to each well. The plate was again sealed, covered and agitated at 800 rpm for 30 min at room temperature then washed as indicated above. Then 50 μl of Streptavidin-R-Phycoerythrin (Prozyme, USA; 5 μg/ml in diluent buffer) was added to each well. The plate was sealed, covered, and agitated at 800 rpm for 30 min at room temperature and washed as indicated above. A 100 μl of 1x Tris-EDTA pH 7.4 was added to each well, and the plate was vortexed for 5 min before reading on the BioRad Bio-Plex 200 instrument following the manufacturer's instructions. The instrument was set up to read beadsets in appropriate regions. A minimum of 60 events per beadset was read, and the median value was calculated. For all samples, the multiplex assay data were corrected by subtracting the background levels.

| **Cytokine** | **Capture antibody** | **Detection antibody** | **Standard** | **Bead** |
| --- | --- | --- | --- | --- |
| **IL-1*β*** | MAb* anti porcine IL-1β/IF2; R&D MAB6811 | Goat anti porcine IL-1*β*/IF2 biotin; R&D BAF681; 0.5 μg/ml | recombinant porc IL-1*β*/IF2; R&D 681-PI-10; 5000 pg/ml | Region 26; BioRad MC10026-01 |
| **IL-6** | Goat anti porcine IL-6; R&D AF686 | Goat anti porcine IL-6 biotin; R&D BAF686; 0.5 μg/ml | Recombinant porcine IL-6; R&D 686-PI-025; 5000 pg/ml | Region 65; BioRad MC10065-01 |
| **IL-8** | MAb anti sheep IL8 (86.9% homology); AbD Serotec MCA1660 | MAb anti porcine CXCL8/IL8 biotin; R&D MAB5351; biotinylated in house; 1:400 dilution | Recombinant porcine IL-8; Kingfisher RP0109S-005; 200 pg/ml | Region 27; BioRad MC10027-01 |
| **IL-10** | MAb anti swine IL-10; Invitrogen ASC0104 | MAb anti swine IL-10 biotin; Invitrogen ASC9109; 0.5 μg/ml | Recombinant swine IL-10; Invitrogen PSC0104; 5000 pg/ml | Region 28; BioRad MC10028-01 |
| **IL-12** | MAb anti porcine IL-12; Kingfisher MA0413S-100 | MAb anti porcine IL12/IL23 p40 biotin; R&D BAM9122; 0.5 μg/ml | Recombinant porcine IL-12; R&D 912-PL-025; 5000 pg/ml | Region 36; BioRad MC10036-01 |
| **IL-13** | Goat anti swine IL-13; Kingfisher PB0094S-100 | Goat anti swine IL-13 biotin; Kingfisher PBB0096S-050; 0.5 μg/ml | Recombinant swine IL-13; Kingfisher RP0007S-005; 5000 pg/ml | Region 52; BioRad MC10052-01 |
| **IL-17A** | Rabbit anti porcine IL-17A; Kingfisher KP0498S-100 | Rabbit anti porcine IL-17A biotin; Kingfisher KPB0499S-050; 0.1 μg/ml | Recombinant porcine IL-17A; Kingfisher RP0128S-005; 2000 pg/ml | Region 62; BioRad MC10062-01 |
| **TGF-*β*** | MAb anti bovine TGF-β1,2,3  R&D MAB1835 | Chicken anti human TGF-*β*1 biotin; R&D BAF240; 0.5 μg/ml | Recombinant human TGF-*β*; R&D 240B002; 2000 pg/ml | Region 55;  BioRad MC10055-01 |
| **TNF** | MAb anti porcine TNF alpha  R&D MAB6902 | Goat anti porcine TNF alpha biotin; R&D BAF690; 0.5 μg/ml | Recombinant porcine TNF alpha; R&D 690PT025; 5000 pg/ml | Region 34;  BioRad MC10034-01 |
| **IFN-γ** | MAb anti porcine IFN-γ; Fisher ENMP700 | MAb anti porcine IFN-γ; Fisher ENPP700; biotinylated in-house; 1:400 dilution | Recombinant porcine IFN-γ; Ceiba Geigy; 2000 pg/ml | Region 43; BioRad MC10043-01 |
| **IFN-α** | MAb anti porcine IFN-α; GeneTex GTX11408 | MAb anti pig IFN-α; PBL 27105-1; biotinylated in house; 1:5000 dilution | Recombinant porcine IFNα; Genentech; 200 pg/ml | Region 45; BioRad MC10045-01 |

*MAb – monoclonal mouse antibodies

**Cortisol ELISA**

To quantify chronical cortisol, hairs were collected at euthanasia, processed and tested using Cortisol ELISA kit (ADI-900-071, Enzo Life Sciences, USA) according to the manufacturer’s instructions. The kit has been validated by the manufacturer for testing porcine samples and has previously been used in pig research [6,8–10]. The assay detection limit was 56.7 pg/ml. Hair samples from the left flank body region were sampled by shaving as close to the skin as possible with electric clippers. Samples were stored in a dark place at -20°C. Hairs were washed with methanol five times (40 μl methanol per mg hair, shaking and inverting for 3 min) in a 50 ml Falcon tubes [11]. After air-drying in a fume hood (RT, three days), 40-60 mg of hairs for each sample was ground into a fine powder using a 5 mm metal bead (ref.: QIAgen 69989) in a 2 ml O-ring tube (25 Hz, 2 x 30 min). For an occasional hair sample that did not grind sufficiently, processing was continued at 25 Hz in 30 s intervals until a fine powder. Upon brief centrifugation, cortisol extraction was done by adding 0.5 ml methanol with slow rotation for 24 h. After centrifugation (15 min at 2000g, RT), the supernatant was transferred to a new 1.5 ml Eppendorf. Methanol was added to hair samples twice more. After vortexing and spinning down all methanol washes were pooled and dried out in a fume hood. Cortisol was concentrated by rinsing the sides of the vial with consecutive methanol washes (0.5 ml, 0.3 ml, 0.15 ml) and repeated drying out of methanol after each wash in a fume hood (+37 ºC). Then cortisol was reconstituted in PBS (200 μl, +4 ºC, 12 h, in the dark place), vortexed, centrifuged (15 min at 2,000g, RT) and tested in ELISA kit. Cortisol concentration for each sample was expressed in nanograms per g (ng/g) of washed and dried hair.

**Histology**

Formalin-fixed brain tissues from piglets were embedded in paraffin, sectioned and stained with hematoxylin and eosin (H&E). Sequential tissue sections were examined under a microscope for calcifications, white and grey matter ratio, and lesions.

**ZIKV RNA *in situ* hybridization (ISH)**

*In situ* hybridization (ISH) was performed with RNAscope 2.5 HD Reagent Kit-BROWN (Advanced Cell Diagnostics, USA) according to the manufacturer's instructions for frozen tissues. The RNAscope V-ZIKV probe targeting ZIKV RNA (ref.: ACD 467771), positive control Sc-PPIB probe targeting pig PPIB transcripts (ref.: ACD 428591), and RNAscope negative control DapB probe (ref.: ACD 310043) were designed and synthesized by Advanced Cell Diagnostics. Tissues were counterstained with hematoxylin and visualized with standard bright-field microscopy. To monitor the specificity of the staining, tissues from mock-exposed and ZIKV-exposed animals were treated with ZIKV-specific and negative control probes, respectively.

**References**

1. Darbellay J, Cox B, Lai K, Delgado-Ortega M, Wheler C, Wilson D, et al. Zika Virus Causes Persistent Infection in Porcine Conceptuses and may Impair Health in Offspring. EBioMedicine. 2017;25: 73–86. doi:10.1016/j.ebiom.2017.09.021

2. Xu MY, Liu SQ, Deng CL, Zhang QY, Zhang B. Detection of Zika virus by SYBR green one-step real-time RT-PCR. J Virol Methods. 2016;236: 93–97. doi:10.1016/j.jviromet.2016.07.014

3. Faye O, Faye O, Dupressoir A, Weidmann M, Ndiaye M, Alpha Sall A. One-step RT-PCR for detection of Zika virus. J Clin Virol. 2008;43: 96–101. doi:10.1016/j.jcv.2008.05.005

4. Nem de Oliveira Souza I, Frost PS, França JV, Nascimento-Viana JB, Neris RLS, Freitas L, et al. Acute and chronic neurological consequences of early-life zika virus infection in mice. Sci Transl Med. 2018;10: eaar2749. doi:10.1126/scitranslmed.aar2749

5. Darbellay J, Lai K, Babiuk S, Berhane Y, Ambagala A, Wheler C, et al. Neonatal pigs are susceptible to experimental Zika virus infection. Emerg Microbes Infect. 2017;6: e6. doi:10.1038/emi.2016.133

6. Trus I, Darbellay J, Huang Y, Gilmour M, Safronetz D, Gerdts V, et al. Persistent Zika virus infection in porcine conceptuses is associated with elevated in utero cortisol levels. Virulence. 2018;9: 1338–1343. doi:10.1080/21505594.2018.1504558

7. Pasternak JA, Ng SH, Käser T, Meurens F, Wilson HL. Grouping Pig-Specific Responses to Mitogen with Similar Responder Animals may Facilitate the Interpretation of Results Obtained in an Out-Bred Animal Model. J Vaccines Vaccin. 2014;05. doi:10.4172/2157-7560.1000242

8. Zupan M, Zanella AJ. Peripheral regulation of stress and fear responses in pigs from tail-biting pens. Rev Bras Zootec. 2017;46: 33–38. doi:10.1590/S1806-92902017000100006

9. Turpin DL, Langendijk P, Chen TY, Lines D, Pluske JR. Intermittent suckling causes a transient increase in cortisol that does not appear to compromise selected measures of pigletwelfare and stress. Animals. 2016;6: 24. doi:10.3390/ani6030024

10. Rault JL, Dunshea FR, Pluske JR. Effects of oxytocin administration on the response of piglets to weaning. Animals. 2015;5: 545–560. doi:10.3390/ani5030371

11. Macbeth BJ, Cattet MRL, Stenhouse GB, Gibeau ML, Janz DM. Hair cortisol concentration as a noninvasive measure of long-term stress in free-ranging grizzly bears (Ursus arctos): considerations with implications for other wildlife. Can J Zool. 2010;88: 935–949. doi:10.1139/z10-057
