# Supplementary figures and images for: Subclinical in utero Zika virus infection is associated with interferon alpha sequelae and sex-specific molecular brain pathology in asymptomatic porcine offspring
Source: PLoS Pathog. 2019 Nov 14;15(11):e1008038. doi: 10.1371/journal.ppat.1008038 (PMC6855438; doi:10.1371/journal.ppat.1008038)

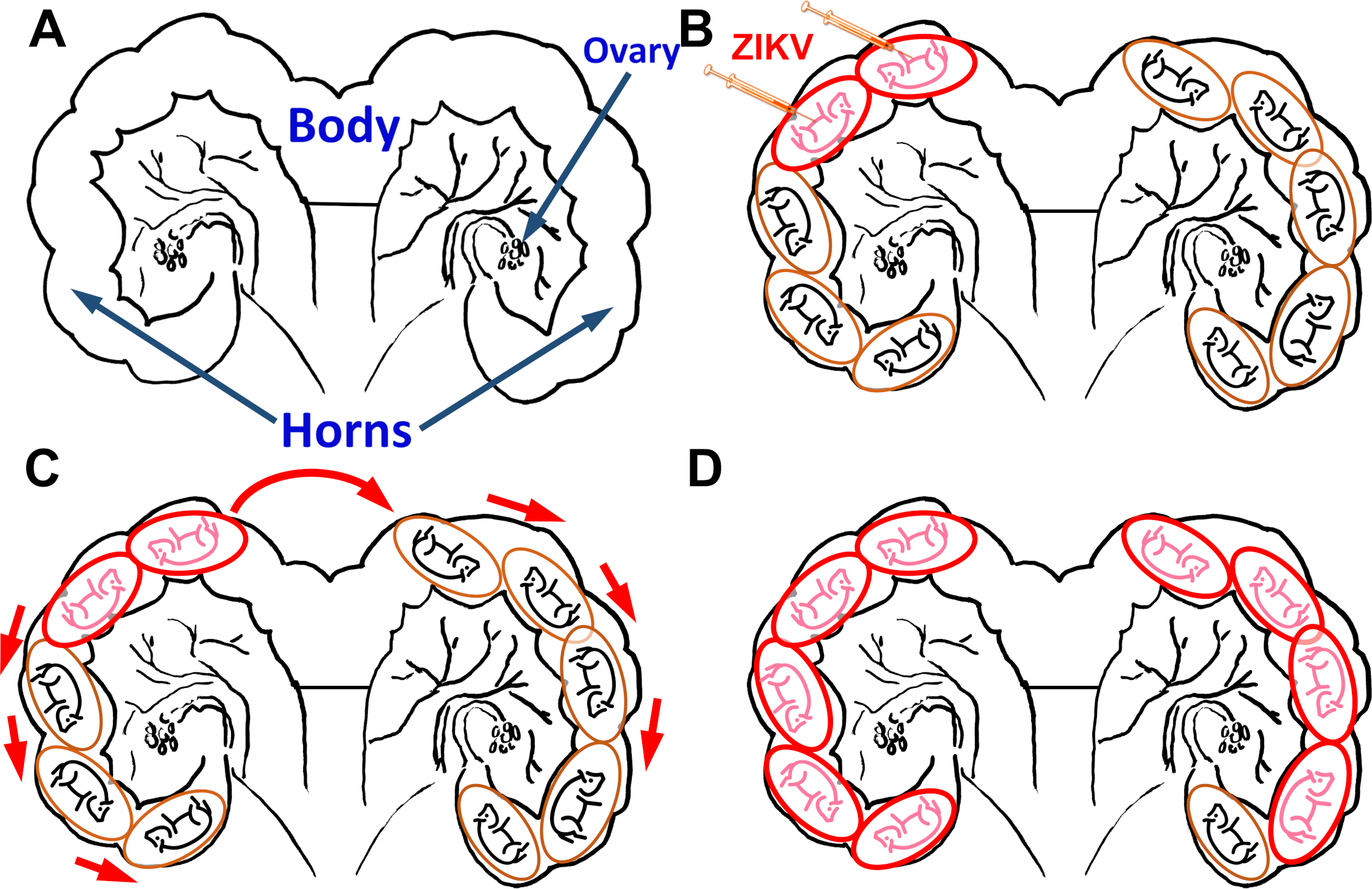

Supplement: S1 Fig — (A) A porcine uterus consists of the uterine body and two horns. (B) Each horn contains multiple fetuses with each fetus possessing individual amniotic membrane and placenta. Two fetuses (highlighted in red) in each pregnant pig were directly inoculated with ZIKV or control media. (C) Afterward, ZIKV spreads between siblings within the horn containing inoculated fetuses and between fetuses in the opposite horn. (D) As a result, most conceptuses (a fetus with fetal membranes) within the uterus are infected. In utero ZIKV infection kinetics and in utero ZIKV tropism in the porcine model is comprehensively described in previous publications [17–19,37]. (TIF) [file ppat.1008038.s002.tif]

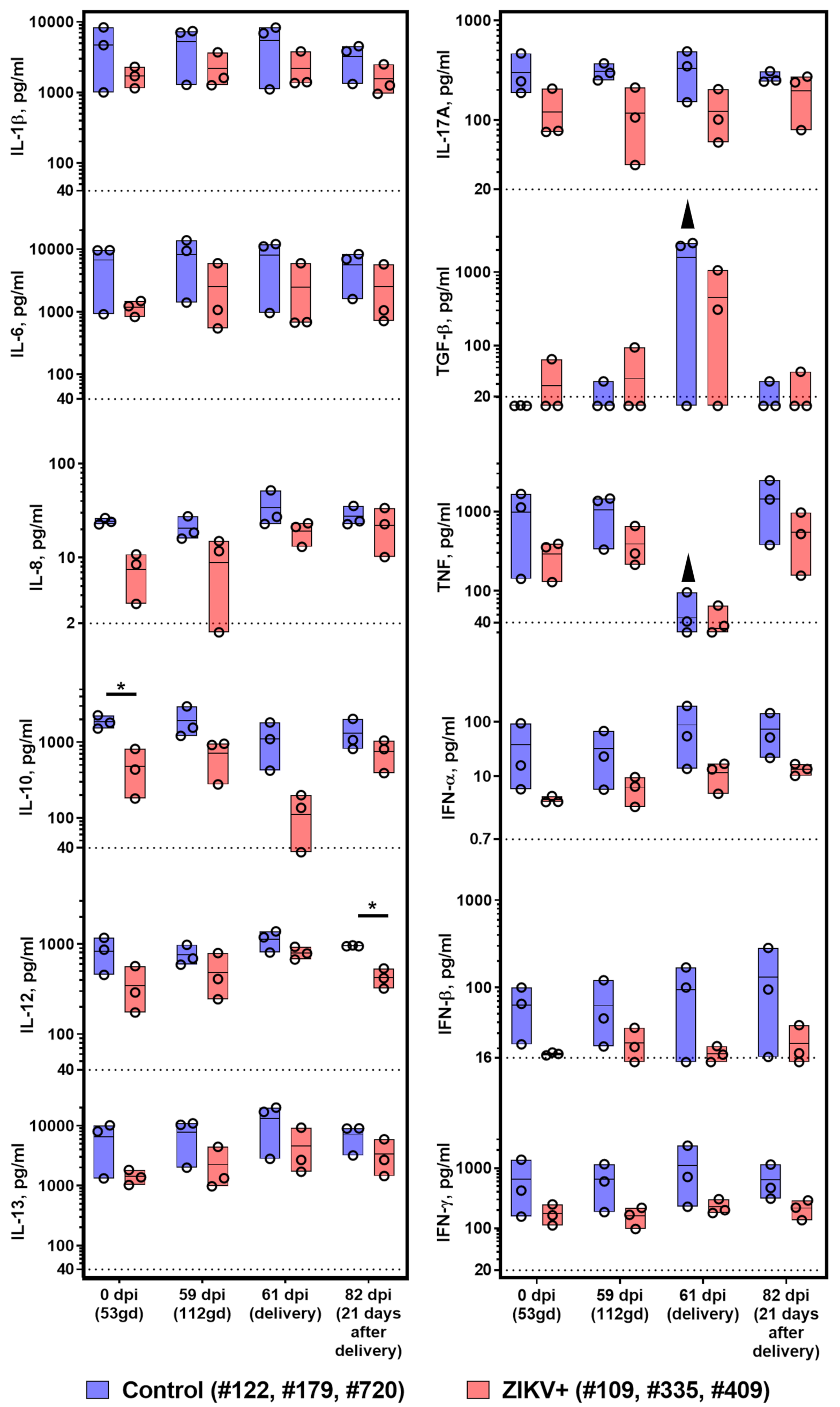

Supplement: S2 Fig — Circles represent data from individual sows. Boxes represent the highest and lowest observations. A horizontal line inside the box is the mean. An asterisk (*) represents a statistically significant difference (P < 0.05) between control and ZIKV groups. An arrowhead (▲) represents statistically significant difference within groups, versus day 0. Dpi–days post-inoculation, gd–gestation days. The dotted line represents LOQ. See raw data in S1D Table for individual values. (TIF) [file ppat.1008038.s003.tif]

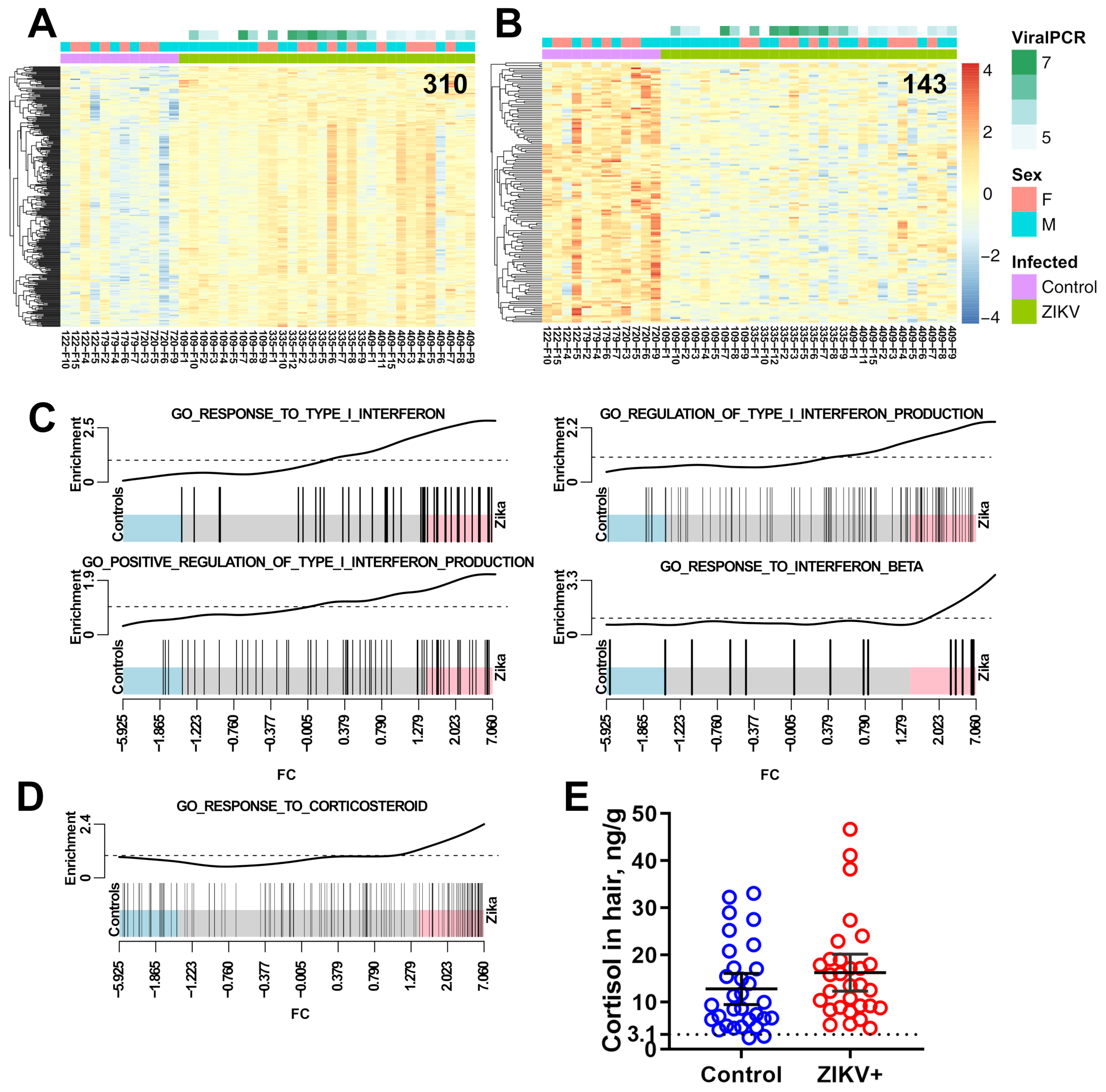

Supplement: S3 Fig — Heatmaps of 310 upregulated (A) and 143 downregulated (B) genes with FDR-adjusted P < 0.05. X and Y axes represent sample identification and genes, respectively. #122, #179, and #720—control litters; #109, #335, #409 –ZIKV litters. See raw data in S2A Table for individual gene values. ViralPCR—represents viral loads in amniotic membranes (S1B Table) (C) Enrichment plots of gene sets of “response to type I interferon” (FDR-adjusted P = 0.0026), “positive regulation of type I interferon production” (FDR-adjusted P = 0.026), “regulation of type I interferon production” (FDR-adjusted P = 0.011) and “response to interferon beta” (FDR-adjusted P = 0.08) GO processes (S2B Table). (D) Enrichment plot of gene sets of “response to corticosteroid” GO process (FDR-adjusted P = 0.03) (S2B Table). (E) Chronic cortisol in offspring hair collected at necropsy. Whiskers denote 95% confidence interval. See raw data in S1F Table for individual values. (TIF) [file ppat.1008038.s004.tif]

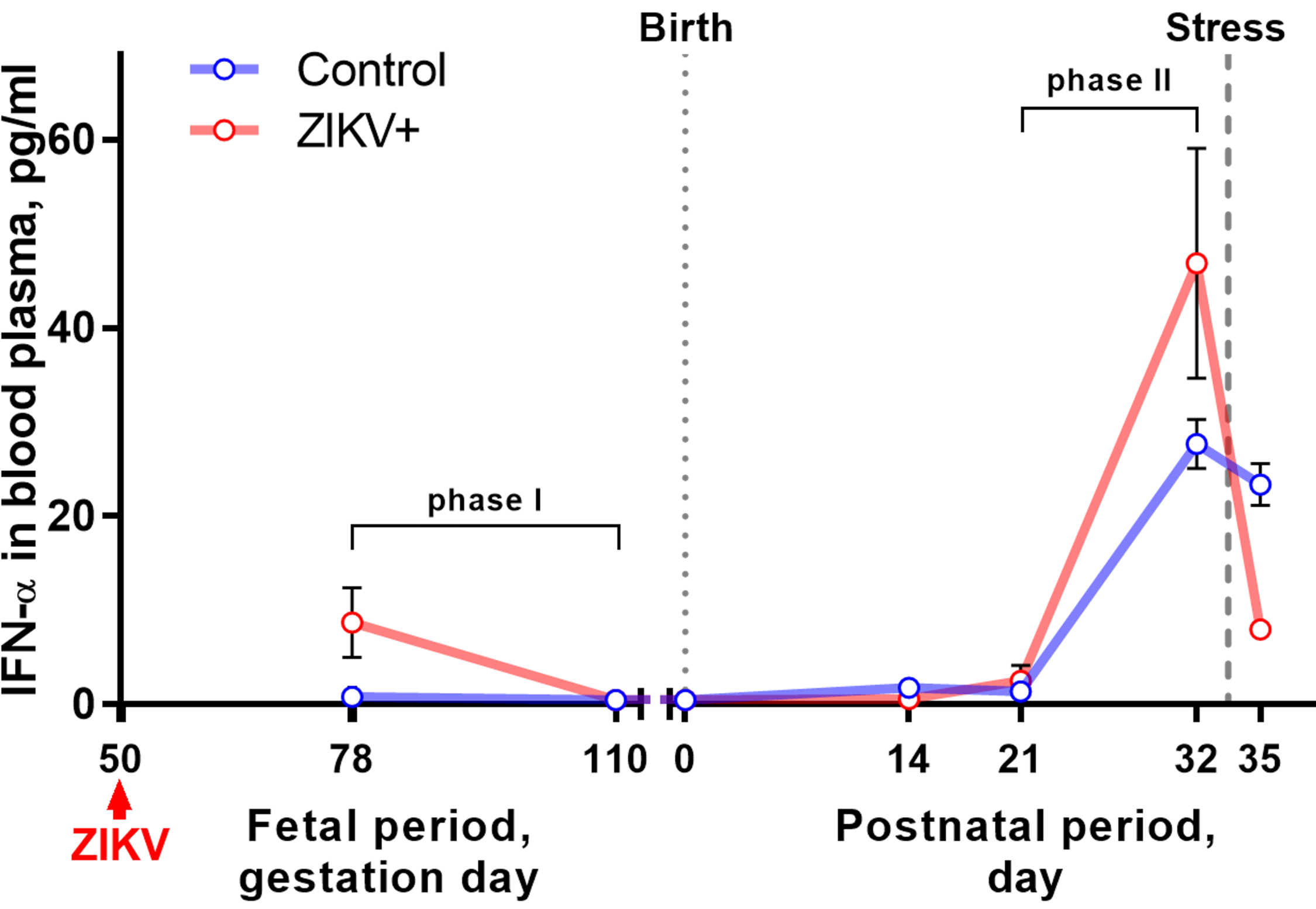

Supplement: S4 Fig — IFN-α levels (M±SE) were measured in the blood plasma of ZIKV-affected and control porcine fetuses and offspring. Data for the fetal period (at 78 gestation days, gd) were compiled from our published study [18], where 53 virus-infected and 22 control fetuses were tested. Data for 110 gd are from study where 14 virus-infected and 16 control fetuses were tested (S1C Table). Elevated IFN-α levels at 78 gd were significantly higher in ZIKV infected fetuses (P = 0.0068, Mann-Whitney test) [18]. (TIF) [file ppat.1008038.s005.tif]
